# Single-cell analysis reveals alterations in cellular composition and cell-cell communication associated with airway inflammation and remodeling in asthma

**DOI:** 10.1186/s12931-024-02706-4

**Published:** 2024-02-05

**Authors:** Xiu Yu, Lifei Li, Bicheng Cai, Wei Zhang, Quan Liu, Nan Li, Xing Shi, Li Yu, Rongchang Chen, Chen Qiu

**Affiliations:** 1 Key Laboratory of Shenzhen Respiratory Diseases, Institute of Shenzhen Respiratory Diseases, Department of Respiratory and Critical Care Medicine, Shenzhen People’s Hospital (The First Affiliated Hospital, Southern University of Science and Technology; The Second Clinical Medical College, Jinan University), Shenzhen, 518020 China; 2 Department of Infectious Diseases, The First Affiliated Hospital (Shenzhen People’s Hospital), School of Medicine, Southern University of Science and Technology, Shenzhen, 518020 China; 3 Department of Biochemistry, Key University Laboratory of Metabolism and Health of Guangdong, School of Medicine, Southern University of Science and Technology, Shenzhen, 518055 China; 4 Longgang Central Hospital of Shenzhen, LongGang District, Shenzhen, 518116 China

**Keywords:** Asthma, Single-cell RNA-sequencing, Cell-cell communication, Airway inflammation, Airway remodeling

## Abstract

**Background:**

Asthma is a heterogeneous disease characterized by airway inflammation and remodeling, whose pathogenetic complexity was associated with abnormal responses of various cell types in the lung. The specific interactions between immune and stromal cells, crucial for asthma pathogenesis, remain unclear. This study aims to determine the key cell types and their pathological mechanisms in asthma through single-cell RNA sequencing (scRNA-seq).

**Methods:**

A 16-week mouse model of house dust mite (HDM) induced asthma (*n* = 3) and controls (*n* = 3) were profiled with scRNA-seq. The cellular composition and gene expression profiles were assessed by bioinformatic analyses, including cell enrichment analysis, trajectory analysis, and Gene Set Enrichment Analysis. Cell-cell communication analysis was employed to investigate the ligand-receptor interactions.

**Results:**

The asthma model results in airway inflammation coupled with airway remodeling and hyperresponsiveness. Single-cell analysis revealed notable changes in cell compositions and heterogeneities associated with airway inflammation and remodeling. GdT17 cells were identified to be a primary cellular source of IL-17, related to inflammatory exacerbation, while a subpopulation of alveolar macrophages exhibited numerous significantly up-regulated genes involved in multiple pathways related to neutrophil activities in asthma. A distinct fibroblast subpopulation, marked by elevated expression levels of numerous contractile genes and their regulators, was observed in increased airway smooth muscle layer by immunofluorescence analysis. Asthmatic stromal-immune cell communication significantly strengthened, particularly involving GdT17 cells, and macrophages interacting with fibroblasts. CXCL12/CXCR4 signaling was remarkedly up-regulated in asthma, predominantly bridging the interaction between fibroblasts and immune cell populations. Fibroblasts and macrophages could jointly interact with various immune cell subpopulations via the CCL8/CCR2 signaling. In particular, fibroblast-macrophage cell circuits played a crucial role in the development of airway inflammation and remodeling through IL1B paracrine signaling.

**Conclusions:**

Our study established a mouse model of asthma that recapitulated key pathological features of asthma. ScRNA-seq analysis revealed the cellular landscape, highlighting key pathological cell populations associated with asthma pathogenesis. Cell-cell communication analysis identified the crucial ligand-receptor interactions contributing to airway inflammation and remodeling. Our findings emphasized the significance of cell-cell communication in bridging the possible causality between airway inflammation and remodeling, providing valuable hints for therapeutic strategies for asthma.

**Supplementary Information:**

The online version contains supplementary material available at 10.1186/s12931-024-02706-4.

## Background

Asthma is a chronic respiratory disease affecting more than 3 billion people worldwide [1]. Approximately 5–10% of asthmatics suffer from severe refractory asthma, these patients are marked with worsening symptoms and exacerbation, they cannot achieve full asthma control or even remain uncontrolled despite daily high-dose inhaled corticosteroids (ICS) plus oral corticosteroids or a second controller [2, 3]. Although these patients account for a small fraction of the asthmatic population, the healthcare resources they consume are over two-thirds of the total costs attributed to asthma [4]. The hallmark pathological features of asthma include airway inflammation, airway remodeling, and airway hyperresponsive. Patients with severe asthma are characterized by irreversible airflow obstruction, airway damage, and a decline in lung function, which are the consequences of airway inflammation and airway remodeling [4]. While the role of airway inflammation has been widely investigated these years [5, 6], most anti-inflammatory drugs (such as mepolizumab) have made promising efficacy in the reduction of inflammation and exacerbation, but do not well address airway remodeling and lung dysfunction [7]. Moreover, it is worth noting that airway remodeling is thought to contribute significantly to irreversible airflow limitation and the disease burden as observed in severe asthmatics. The extent of airway remodeling correlates positively with the severity of asthma. Therefore, preventing or even reversing airway remodeling will progress toward a potential cure for asthma, airway remodeling-characterized refractory asthma in particular.

Airway remodeling refers to broad structural changes consisting of increased airway smooth muscle (ASM) mass, basement membrane thickening, enhanced collagen deposition, subepithelial fibrosis, angiogenesis, goblet cell and mucus gland hyperplasia [8, 9]. The inception and progression of airway modeling in asthma were a complex and sustained process affected by numerous factors, and the underlying mechanisms remain largely unclear. Growing evidence suggests that airway remodeling can be a secondary event to airway inflammation, as asthmatic patients can benefit from steroid treatment whose airway inflammation as well as considerable airway remodeling were relieved [10–12]. In addition, another study has demonstrated that airway remodeling, particularly the increased airway smooth muscle mass, can occur in the absence of inflammation in certain cases [13]. Given the pathological and phenotypic heterogeneity inherent in asthma, driven by diverse molecular mechanisms [14, 15], it is plausible that both inflammation-dependent and inflammation-independent airway remodeling may coexist. Nonetheless, the causality and relationship between airway inflammation and airway remodeling in asthma remain to be fully elucidated.

Currently, 4-week and 8-week murine models of house dust mites (HDM) induced allergic inflammation were commonly used to investigate the underlying pathological mechanisms and factors contributing to airway inflammation and partial airway remodeling in asthma [16, 17]. Considering airway remodeling as a result of long-term disruption and modification in airway architecture, our study aimed to establish a 16-week mouse model of HDM-induced chronic asthma that captures features of airway inflammation, and airway remodeling, hyperresponsiveness, infinitely close to asthmatics. Of note, asthma development is an elaborate multistep process modulated by numerous cells and their interactions. We further employed single-cell RNA sequencing (scRNA-seq) for a comprehensive and high-resolution analysis of the complete lung cell atlas, seeking to decipher cell heterogeneity and transcriptional profiles relevant to asthma pathogenesis. Our data pinpointed several pathological subpopulations intricately associated with inflammation and structural changes, shedding light on their cellular functions and the underlying molecular mechanisms contributing to asthma pathology.

Here, we refined our knowledge of the crucial roles of GdT17 cells and myofibroblast dominantly associated with inflammation exacerbation and smooth muscle mass thickening, respectively, which had been assessed by studies employing the HDM model since 2004 [18]. To identify the key players and the precise mechanisms linking airway inflammation and airway remodeling, we utilized CellChat and Nichenet. These tools enabled us to construct a dynamic map of the cell-cell communication network, with a particular focus on the interactions between structural and immune cells. Our study identified specific ligand-receptor interactions contributing to airway inflammation and remodeling. We identified that the interaction between myofibroblasts and interstitial macrophages emerges as a central hub in the cell-cell communication network, playing a crucial role in promoting the pathogenesis of asthma. This emphasizes the significance of this cell-cell interaction in the pathology of diseases, as described in other studies [19, 20]. Overall, our study provides more insights into stromal-immune cell communications and pivotal ligand-receptor pairs responsible for connecting airway inflammation and airway remodeling, which may offer new strategies for novel therapies against asthma, particularly for refractory asthma characterized by airway remodeling.

## Methods

### Establishment of HDM-induced asthma model

BALB/c female mice at 6 weeks of age were obtained from Yaokang Biotechnology Co., Ltd. (Guangzhou, China). HDM was purchased from Greer Laboratories (Beijing, China, Cat# XPB91D3A25, Lot# 381,017). A bottle of HDM (25 mL/vial) includes protein (42.93 mg/vial) and endotoxin (422.5 EU/vial). Briefly, 42.93 mg HDM protein was dissolved in 17.172 ml 0.9% saline solution to get the HDM final solution (2.5 µg/µl). BALB/c female mice were sensitized by nasal inhalation of 10 µl HDM solution five times a week for 16 weeks. The lung function of conscious mice was determined at week 17 using FinePointe™ Non-Invasive Airway Mechanics chambers. Mice were anesthetized with an intraperitoneal injection of pentobarbital (50 mg/kg) and then bronchoalveolar lavage fluid (BALF) and lung tissues of anesthetized mice were harvested on the same day and all kept at -80 ºC refrigerator for follow-up experiments.

### Lung function determination

The specific airway resistance (sRaw) of conscious mice was detected and analyzed by FinePointe™ Non-Invasive Airway Mechanics chambers (Buxco Electronics, Inc., Wilmington, North Carolina). This used double-flow plethysmography that calculated sRaw by analyzing breathing patterns at nasal and thoracic airflows. For the determination of sRaw in mice, inhalations of saline and methacholine were administered. Ordinally, 0 (PBS), 3.125, and 6.25 mg/mL of methacholine were delivered into the nasal cavity for 30 s in a dose-response manner by aerosols. All measurements were processed in an air-conditioned environment controlled for temperature (22–23℃) and humidity (50–60%).

### Determination of lung pathological change by hematoxylin-eosin (H&E), periodic acid-schiff (PAS), masson staining and immunohistochemistry

Briefly, the lung tissues were fixed with 4% paraformaldehyde, embedded in paraffin, then cut into 4 μm thickness sections, and stained with H&E staining kit (#G1120), PAS staining kit (#G1280) and Masson staining kit (#G1340) purchased from Solarbio Life Sciences for detection of inflammatory cells, determination of mucus-secreting cells and collagen fiber deposition with light microscopy. For immunohistochemical staining, the lung tissues were exposed to H2O2 to block endogenous peroxidase and then incubated with primary antibodies against α-SMA overnight at 4 °C. A horseradish peroxidase-streptavidin detection system (Maixin Biotech, Fuzhou, China) was employed to detect immunoactivity followed by counterstaining with hematoxylin. The scores of indicated indexes were analyzed by Image Pro Plus 8.0 and Caseviewer 2.4.0.119028, according to previous research [21–23]. Sections were performed in at least five different fields for each lung section and were assessed in a random blinded manner by two independent investigators, as shown in Table S1.

### Determination of α-SMA, POSTN and CHRM2 expression in lung tissues with immunofluorescence

The lung tissues were washed with cold PBS, and permeabilized with 0.3% Triton X-100 in PBS for 20 min at room temperature. After blocking with 5% BSA, lung tissues were incubated with the indicated primary antibodies against POSTN, CHRM2 and α-SMA (1:200) overnight at 4 °C, washed by cold PBS, and then incubated with Alexa Fluor™ 488-conjugated secondary antibody (1:1000) in PBS for 2 h. Images were taken by laser scanning confocal microscopy (Leica, Germany).

### Sample preparation and single-cell RNA sequencing

Mice were sacrificed and lung tissues were obtained from the asthma (n = 3) and control (n = 3) groups. Following a wash with sterile 1× PBS, tissues was cut into small pieces. To dissociate tissues into single cells, the tissues were digested in dissociation solution (1 mg/mL collagenase D, 120 units/mL DNase I and 2 mg/mL papain) at 37°C for 30 min. The resulting single-cell suspensions were filtered through a 70µm cell strainer. Cell enumeration was conducted using 0.2% trypan blue exclusion. Prepared samples were performed according to the instruction manual of the Chromium Single Cell 3’ Reagent Kits v3scRNA-seq by Jiayin Biotechnology Ltd. (Shanghai, China).

### ScRNA-seq library construction with 10x genomics platform

Cellular suspensions were loaded on the Chromiu Controller (10x Genomics, Pleasanton) to generate Gel-beads in emulsions. Barcoded sequencing libraries were conducted following the instruction manual of the Chromium Single Cell 3’ Reagent Kits v3 (10x Genomics). Following the library preparation, the sequencing was performed with paired-end sequencing of 150nt at each end on one lane of Nova-Seq 6000 per sample. The reads were mapped onto the Mm10 genome https://support.10xgenomics.com/single-cell-atac/software/release-notes/references#mm10-2020-A using a standard CellRanger pipeline https://support.10xgenomics.com/single-cell-gene-expression/software/pipelines/latest/using/tutorial_ov.

### scRNA-seq data statistical analysis

Raw data of each sample was firstly demultiplexed and aligned to mm10 reference genome, then the unique molecular identifier (UMI) counts were quantified with 10X Genomics Cell Ranger pipeline (v2.1.1, 10X Genomics). Quality control, integration and clustering were performed with Seurat version 4.0.1 [24]. Loading filtered expression matrices of each sample into R as seurat object, we filtered out the cells that expressed less than 200 genes, we also removed genes detected in less than 3 cells. Cells with a percentage (< 20%) of UMIs mapped to mitochondrial genes were retained. SCT transform was applied to normalize each sample and select highest variable genes, this step was useful to regress out effects of cell cycle and cell stress [25]. Select Integration Features, Prep SCT Integration, Find Integration Anchors, and Integrate Data functions with default options were further performed on SCT transform-normalized data. Run PCA was used on the top 3000 variable genes and top 30PCs, then the data was clustered with dims = 1:20 and resolution = 1.2 based on the PC Elbow Plot. Cell clusters were visualized using Uniform Manifold Approximation and Projection (UMAP). Doublet Finder was used to estimate doublets and implemented with Seurat to plot them in the same UMAP space with a doublet rate of 1% [26]. To identify the differential expression genes (DEGs), we used Model-based Analysis of Single-cell Transcriptomics test [27] with genes detected in a minimum of 10% of all cells, a minimum of 0.2 average log fold-change, and a minimum of 0.05 adjusted p-value implemented in the Findmarker function by Seurat. The major cell types (immune, epithelial, endothelial, stromal and mesothelial cell) were annotated by signature gene markers PTPRC, EPCAM, PECAM1, COL1A1 and MSLN, respectively [28]. We further clustered each major cell type into sub-clusters with same data process as mentioned above, PTPRC + cells, which were likely to be rare doublets and contaminants, were removed from all non-immune cells. We next re-clustered the retained cells without integration, PCA and UMAP were performed with npcs = 30, dims = 1:20 and resolution = 1.2. Cell types were annotated by canonical gene markers as shown in Table S2 [28].

### Pathway and functional analysis

Gene Set Enrichment Analysis (GSEA), pathway and functional analysis were performed with the R package fgsea v1.16.0 and cluster Profiler v3.18.1. We focus on the ontologies loading from R package msigdbr v7.4.1, including KEGG Pathways, Reactome Pathways, Biological Processes and the MSigDB Hallmark Gene Sets. Significantly enriched terms were those with an FDR ≤ 0.05, the normalized enrichment score was used to assess whether the term was associated with up-regulated or down-regulated genes in a given condition. Duplicated terms were removed by custom R script.

### Construction of the cellular trajectory analysis

R package Monocle (v2.18.0) [29] was applied to construct cellular lineage trajectory along the pseudotime. Differential expression genes were identified by “differential Gene Test” function. Top 2500 most variable genes with the lowest q value were selected and used for subsequent analysis. Data was reduced and visualized into two-dimensional space with method “DDR tree” in “reduce Dimension” function. Cells were ordered along the cellular trajectory, Heatmap was used to visualize the genes detected by BEAM function that contributed most to cell developmental paths. These genes were further employed for KEGG and GO biological process enrichment analysis.

### RNA velocity analysis

Splice and un-spliced counts matrices were constructed by Velocyto [30] pipeline based on “Cell Ranger” output files; we applied default parameters unless stated otherwise. Loom files for each sample of normal and asthma groups were both created and merged. Top 3000 most variable genes were retained, and spliced counts were at least 100. After data normalization and scale, cell clusters were embedded with the velocity streams based on the ratio of spliced and un-spliced, and projected onto UMAP plot for visualization.

### Cell-cell communication analysis

To identify cell-cell communication in lung tissue, CellChat (v1.1.3), a public repository of ligand-receptor interactions, was employed [31]. CellChat object was first generated from Seurat object with “CellChat” function. “Identify Over Expressed Genes” function was employed to identify over-expressed ligands and/or receptors and over-expressed ligand-receptor interactions in specific sample status. Communication probability and inferring cell-cell communication network were predicted by “CommunProb” and “computeCommunProb Pathway” functions based on the calculation of the average gene expression per cell status. Cell interaction with a p-value < 0.05 was defined as significance. All graphs to visualize the cell-cell communication network can be created by various functions implemented in CellChat. We next employed R package “NicheNet” [32] to predict sender cells’ ligand-downstream target genes in receiver cells by integrating gene expression profiles with prior knowledge of signaling pathways and gene regulatory networks. To characterize the communication network between fibroblast and macrophage, we specifically defined interstitial macrophages (Int Macro) and alveolar macrophages (Alv Macro) and col14a1 + Fib and col14a1 + Fib/Myofib to be mutual sender/niche cells and receiver/target cells. Top 30 ligands with the highest ligand activity and corresponding target genes and receptors were visualized in the linkage heatmap. The expression level of top ligands and comparison between normal and asthma groups were exhibited as well. Target genes were subsequently used for performing pathway and functional analysis.

### Cell enrichment analysis

Enrichment analysis was conducted to examine the enrichment: (i) if immune cell and/or non-immune was enriched in asthma group; (ii) if any cell type out of 34 cell clusters was enriched in asthma group; and (iii) if any cell sub-type in re-cluster of major cell types was enriched in asthma group. The significance was evaluated using single-sided Fisher’s exact tests, FDR represented for adjusted P value after multiple tests.

### Statistical analysis

All independent repeat experiments were performed at least in triplicate. Data are presented as the mean ± standard deviation. Results were interpreted using One-way analysis of variance with a Dunnett’s *post hoc* test (GraphPad Prism 5.0 software). *P*-values < 0.05 were considered statistically significant.

## Results

### A 16-week model of HDM-induced allergic asthma characterized by both airway inflammation and remodeling

**Fig. 1:**
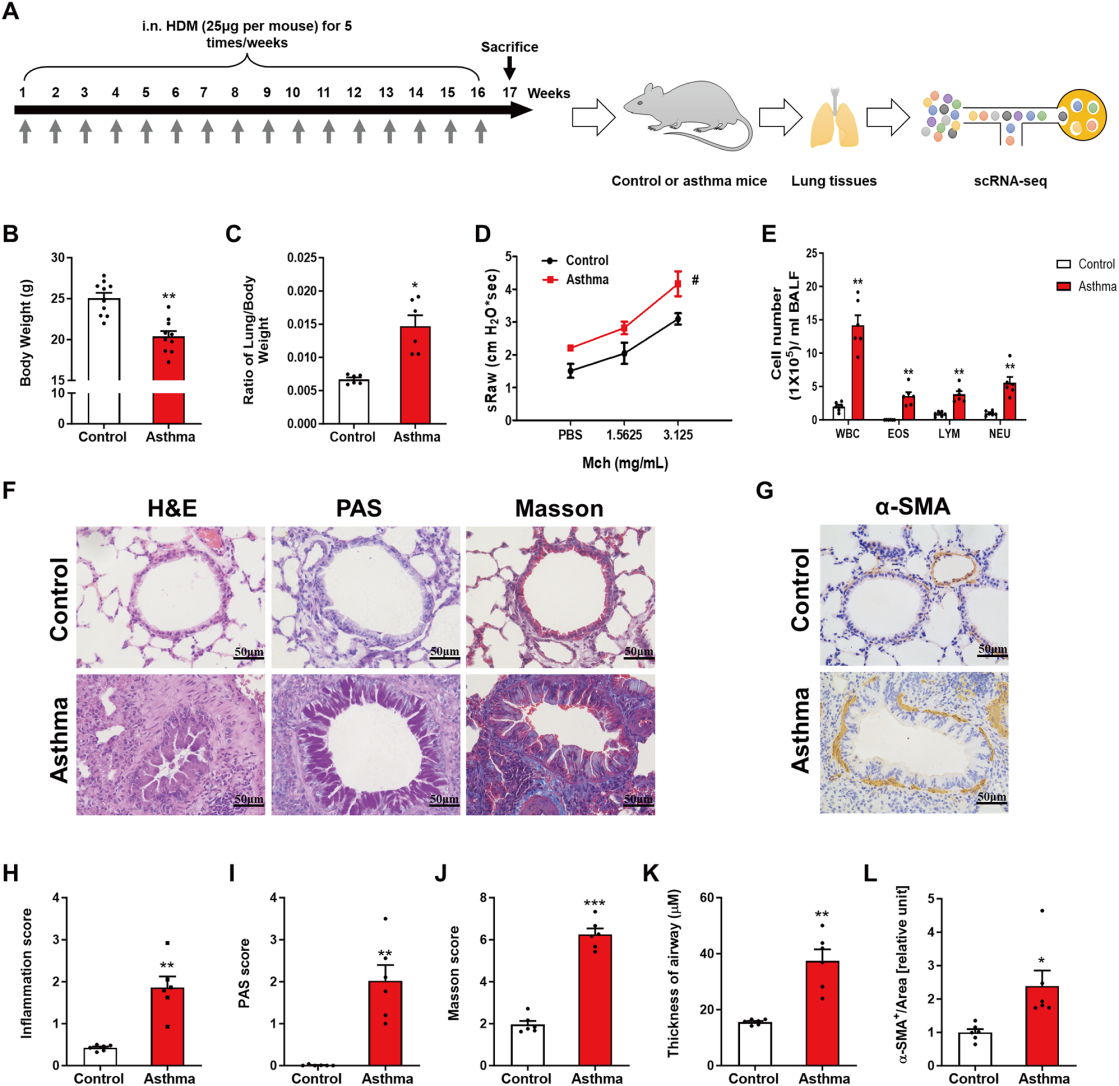
A 16-week model of HDM-induced asthma results in airway inflammation, airway remodeling and hyperresponsiveness. (**A**) Schematic diagram of established asthma model and subsequent scRNA-seq analysis. (**B**) Body weight of asthma group was markedly reduced. (**C**) Ratio of lung/body weight was significantly reduced in asthma group. (**D**) sRaw of asthma group was increased compared to controls. (**E**) The number of total cells, eosinophils, lymphocytes and neutrophils in BALF were compared between and asthma group and controls. (**F**) Comparison of H&E, PAS and Masson staining in mice lung section of asthma group and controls. (**G**) The expression level of α-SMA was significantly increased in lung tissues of asthma group. (**H**) Inflammation score in asthma group was significantly increased. (**I**) PAS score in asthma group was significantly increased compared with controls. (**J**) Masson’s score of asthma group was markedly increased. (**K**) Thickness of airway wall in asthma group was significantly increased compared to controls. (**L**) α-SMA positive areas of asthma group were remarkably increased. The values are shown as the mean ± SD (*n* = 6). ##*P* < 0.01 vs. and #*P* < 0.05. Scale bar, 50 μm. The values are shown as the mean ± SD (*n* = 6). ^***^P < 0.001νs., ^**^P < 0.01 νs. and ^*^P < 0.05νs. Control group

We established a 16-week mouse model of asthma induced by HDM, followed by lung specimen extraction and immediately processed for scRNA-seq, the framework of our study was illustrated in Fig. 1A. Mice were intranasally administered 25 µg HDM for 5 consecutive days weekly for up to 16 weeks [18, 33]. Compared with normal control group (NC), the asthma group (AS) exhibited a marked reduction in body weight and a significant increase in the ratio of the lung to body weight (Fig. 1B-C, *P* < 0.05, Student t test). Additionally, sRaw was notably enhanced in AS (Fig. 1D, *P* < 0.05, Student t-test). We observed a significant increase in the total number of eosinophil, macrophage, neutrophil and lymphocyte populations in BALF of AS (Fig. 1E, *P* < 0.05, Student t-test), indicating inflammation response triggered by HDM. We further performed a histological analysis to identify the pathological characteristics in AS. When comparing with NC, we observed a remarkable inflammatory cell infiltration around the airway and substantial mucus hyperproduction and goblet cell hyperplasia as well as increased collagen deposition and the elevated expression level of α-SMA (ACTA2) around the airway areas in AS (Fig. 1F-G). The statistical significance was shown in Fig. 1H-J (see method). Besides, the airway wall thickness in AS was significantly increased than that of NC (see method), aligning with elevated expression level of ACTA2 (Fig. 1K-L). These observations supported the successful establishment of AS model, which was characterized by airway inflammation, airway remodeling, and hyperresponsiveness.

### Divergent cellular composition and expression profiles across the entire lung in NC and AS

**Fig. 2:**
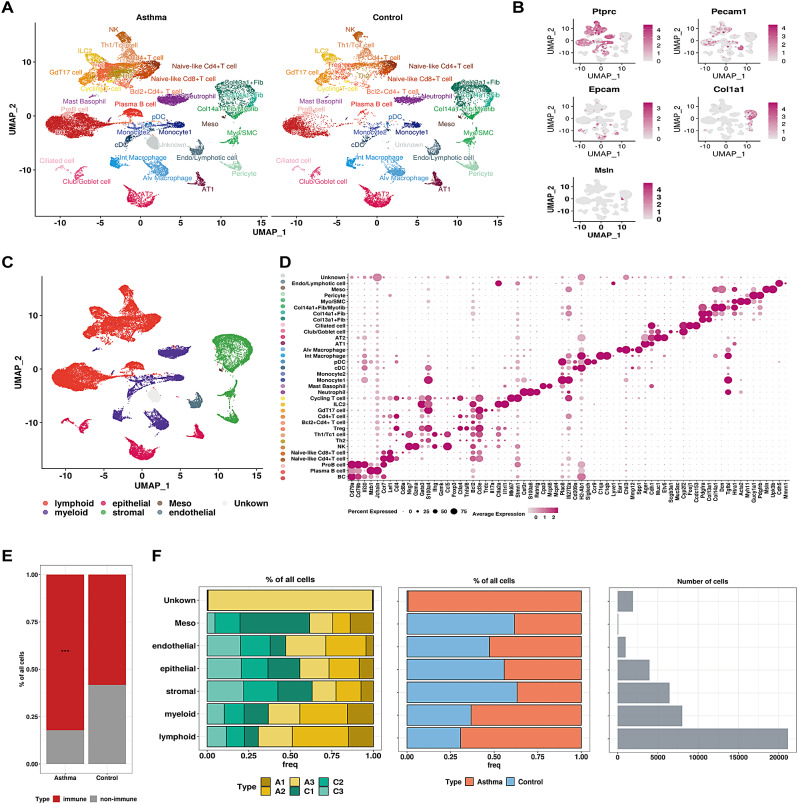
Map of the cellular composition of lungs in the NC and AS groups. (**A**) UMAP plot of scRNA-seq data from normal (*n* = 3) and asthma (*n* = 3) identified 34 cell clusters. (**B**) Feature plot of markers defining immune, epithelial, endothelial, mesenchymal and mesothelial populations. (**C**) UMAP plot illustrating major cell populations identified by distinct markers, with each population represented by different colors. (**D**) Dot plot of selected marker genes for each cell cluster. (**E**) Comparison of immune and non-immune cell proportion in normal and asthma, the significance of enrichment is computed by Fisher’s exact test with asterisks *** representing FDR (false discovery rate) < 0.01. (**F**) Left: Stacked bar plot showing relative cell proportion in each sample across major cell populations (A1-A3: asthma, C1-C3: normal); middle: the percentage of cells derived from normal and asthma group within different major cell types; right: the total cell number in each cell cluster

Next, we generated scRNA-seq profiles using the 10x Genomics platform for six lungs from NC and AS groups with three replicates each group. With standard data process [28], a total of 42,561 cells (AS = 26,297, NC = 16,264) passed quality control filtering were retained for subsequent analysis. UMAP-clustering analysis identified 34 cell clusters, which were annotated using established marker genes (Fig. 2A-B, Fig. S1A, Table S2). These clusters corresponded to six major cell types: lymphoid (*n* = 21,377) and myeloid (*n* = 7,808) marked by PTPRC; epithelium (*n* = 3,945) detected by EPCAM; mesothelium (*n* = 86) with high expression in MSLN; endothelium (*n* = 993) identified by PECAM1; stromal (*n* = 6452) exhibited high COL1A1 expression; and an undefined sub-cluster labeled as “Unknown” (*n* = 1,900) (Fig. 2C-D).

We observed a significant increase in the proportion of total immune cells in the AS group compared with NC (Fig. 2E, Fisher’s exact test, *P* < 2.2e-16), indicating a predominance of immune activation in asthma model. Although individual variations in the cell composition of the six mice from NC and AS groups, both the number and proportion of lymphoid and myeloid cells sharply increased in the AS (Fig. 2F). The transcriptomic profile of the “unknown” sub-cluster appeared to belong to Plasma B cell and was primarily present in one mouse (A3), likely due to biological variability and not subject to further analysis. Intriguingly, B cells, Plasma B cells, Th2, Tregs and neutrophils were the most abundant cell clusters in AS (Fig. S1B), consistent with their vital roles in the inflammatory response in asthma. DEGs between AS and NC groups revealed subpopulations of macrophages and stromal cells with a higher number of DEGs compared to other cell clusters, suggesting that the dysfunction of these DEGs maybe more responsive to HDM effect (Fig. S1C). These observations indicated that immune cell infiltration was appropriately triggered and promoted by allergen. We have successfully constructed a detailed map of lung cells in NC and AS mice, laying the foundation for subsequent research.

### Interactions between stromal cells and immune cells served as a central communication hub in asthma

**Fig. 3:**
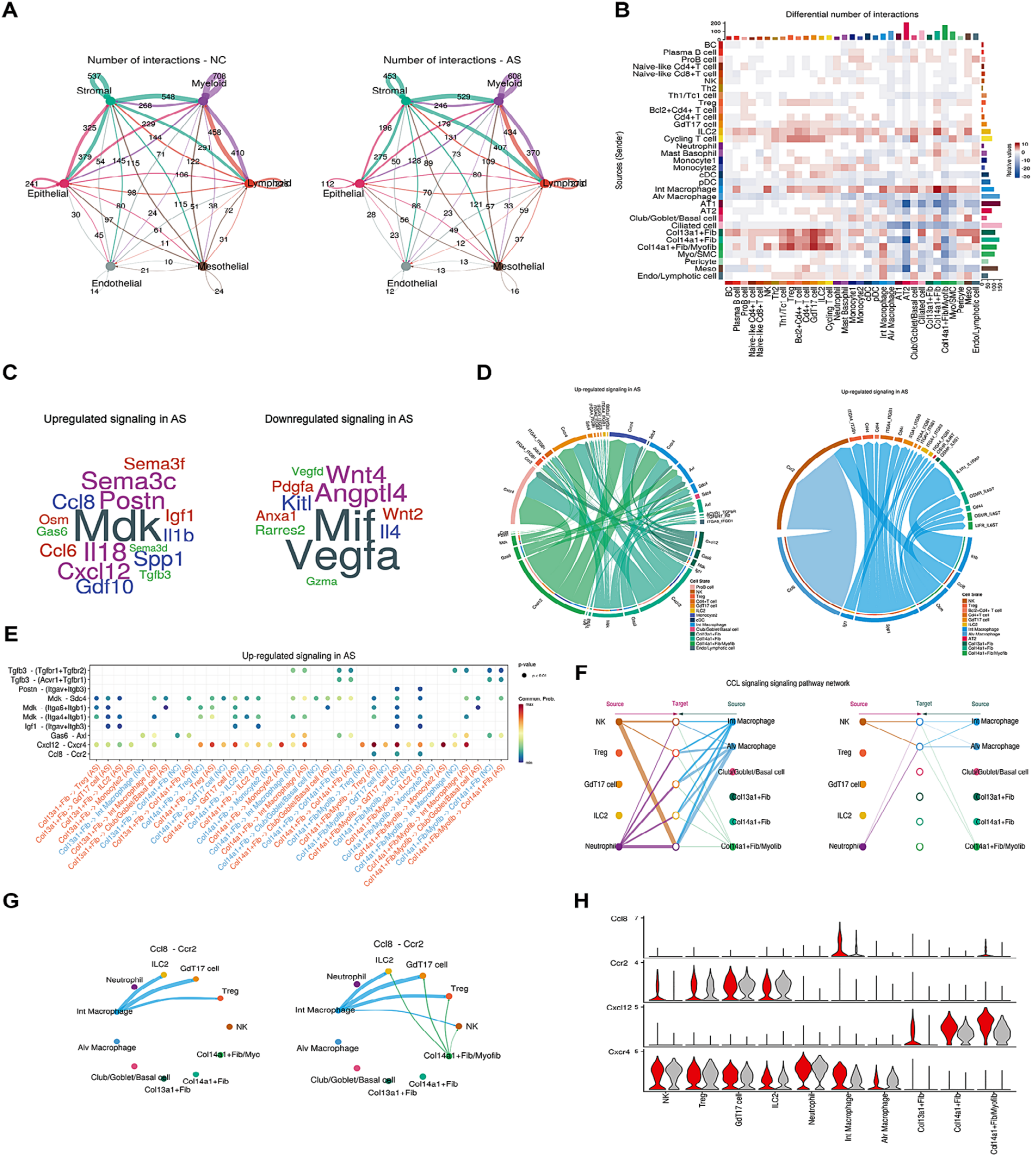
Cellchat analysis illustrating cell-cell interaction specific to asthma. (**A**) Circle plot showing the number of ligand-receptor interactions between pairwise cell populations among the six major cell populations in NC and AS groups. The strength of ligand-receptor (L-R) interactions between cell population pairs was visualized with the number of L-R pairs labeled, and edge width was proportional to the number of L-R pairs. (**B**) The heatmap depicted the different number of pairwise cell interactions between AS and NC groups where top colored bar plot denoted the sum of incoming signaling and right colored bar plot represented the sum of outgoing signaling in each cell cluster. The color bars in red and blue respectively denoted an increase and decrease in the number of interactions in AS compared to NC group. (**C**) Wordcloud visualizing the up-regulated and down-regulated ligands in asthma. Word size indicates the extent of enrichment in asthma or control group. (**D**) Chord diagram illustrated ligand-receptor pairs and their weight contributing to the signaling from fibroblast subpopulations and macrophage subpopulations to the selected signaling receiving cells. (**E**) Comparison of the significant ligand-receptor pairs between AS and NC groups, which contribute to the signaling pathways from fibroblast subpopulations to the selected cells described in Fig D. Dot color represented communication probability and dot size denoted p-value. (**F**) Hierarchical plot deciphering cell communication network of CCL signaling in AS group. Left and right panels showed autocrine and paracrine signaling to selected signaling receiving cells and sender cells. The width of edge reflected the communication probability. (**G**). Circle plot showing inferred CCL8-CCR2 pair in NC (left) and AS group (right). (**H**). Violin plots displaying expression level of selected genes in major senders and receivers in NC (grey) and AS group (red)

To characterize the cell-cell communication specific to asthma, we utilized CellChat tool to explore putative ligand-receptor interactions from our high-resolution scRNA-seq data. Stromal, lymphoid and myeloid populations emerged as the predominant communication ‘hub’ in both groups, despite the number and strength of cell interactions being remarkably distinct (Fig. 3A). Compared to NC, all subpopulations of fibroblast and Int Macro showed more outgoing interactions in AS (Fig. 3B), suggesting their prominent roles in the intercellular communication of asthma. In particular, Int Macro-fibroblast interactions and interactions between fibroblast and GdT17 cells as well as ILC2 were all remarkably strengthened in AS. Conversely, the epithelial population exhibited fewer interactions with other cell populations in AS versus NC. Notably, these observations were consistent with a prior scRNA-seq study in asthma, that is, reduced epithelial-stromal interactions were also observed in human asthma compared to controls [34].

To delineate the AS-specific communication architecture, Cellchat identified 27 significantly up-regulated ligand-receptor pairs from 12 signaling pathways, including ligands MDK, CXCL12, POSTN, IL1B, CCL8, and IGF1 (Fig. 3C). CXCL12 has been shown to participate in inflammatory response and airway remodeling in both OVA- and HDM-induced asthma models [35, 36], while POSTN was confirmed to be upregulated in epithelial and subepithelial layers in bronchial biopsies of asthmatics [37]. In addition, IL1B was known to contribute to Th2 inflammation via activation and recruitment of multiple inflammatory cells such as eosinophils and mast cells in asthma [38]. Of note, MDK was known to play an important role in cell growth, proliferation, migration as well as angiogenesis, which was observed as an important signaling pathway for interaction between macrophages and endothelium in a monkey model of asthma [39]. Yet its role in the pathogenesis of asthma was unclear and warrants further investigation to unravel its functions in asthma. Moreover, 20 ligand-receptor pairs from 11 signaling pathways were significantly down-regulated in AS, such as MIF, VEGFA, and WNT4. Downregulation of VEGFA might explain association between the reduction of VEGFA and increased asthmatic mucous metaplasia, as reported in a previous study [40]. In our examination of ligand-receptor pairs within the active signaling pathways in asthma, we observed that subpopulations of fibroblast and macrophage could interact with their major receivers through various paracrine signaling, and fibroblast further demonstrated significant autocrine signaling (Fig. 3D). To investigate the up-regulated fibroblast-to-immune cell signaling, we identified 10 ligand-receptor pairs implicating TGFB3, MDK, IGF1, CXCL12, and CXCL12-CXCR4 demonstrated the most pronounced enhancement in the AS (Fig. 3E). Notably, Int Macro appeared to be new sender of SPP1 to different cell types in AS (Fig. S2A-B).

Putative fibroblast (Fib) (Col13a1 + Fib and Col14a1 + Fib) and myofibroblast (Myofib) (Col14a1 + Fib/Myofib) could specifically interact with GdT17 cell and ILC2 via IGF1 and POSTN signaling pathway in AS, respectively (Fig. 3E, Fig. S2C). Of note, Col14a1 + Fib/Myofib in AS were identified as new senders of CCL8, together with Int Macro, targeting NK cells, Treg cells, GdT17 cells, and ILC2 through the CCL8-CCR2 pair (Fig. 3F-G), indicating a potential cooperative relationship between fibroblast and interstitial macrophages in cell-cell interactions. The ligand-receptor pairs involved in these pathways showed distinct expression profiles between AS and NC (Fig. 3H, Fig. S2D). In AS, Int Macro showed high expression levels in SDC4, IGF1, CCL8 and CXCL4, while CXCL12 and IGF1 were up-regulated in all subpopulations of fibroblast and POSTN was uniquely highly expressed in Col14a1 + Fib/Myofib. These findings suggested that the specific enhancement of stromal-immune cell communication was likely associated with airway inflammation and airway remodeling in the asthma model.

### GdT17 cells emerged as the primary source of IL-17 associated with inflammatory response and asthma severity

**Fig. 4:**
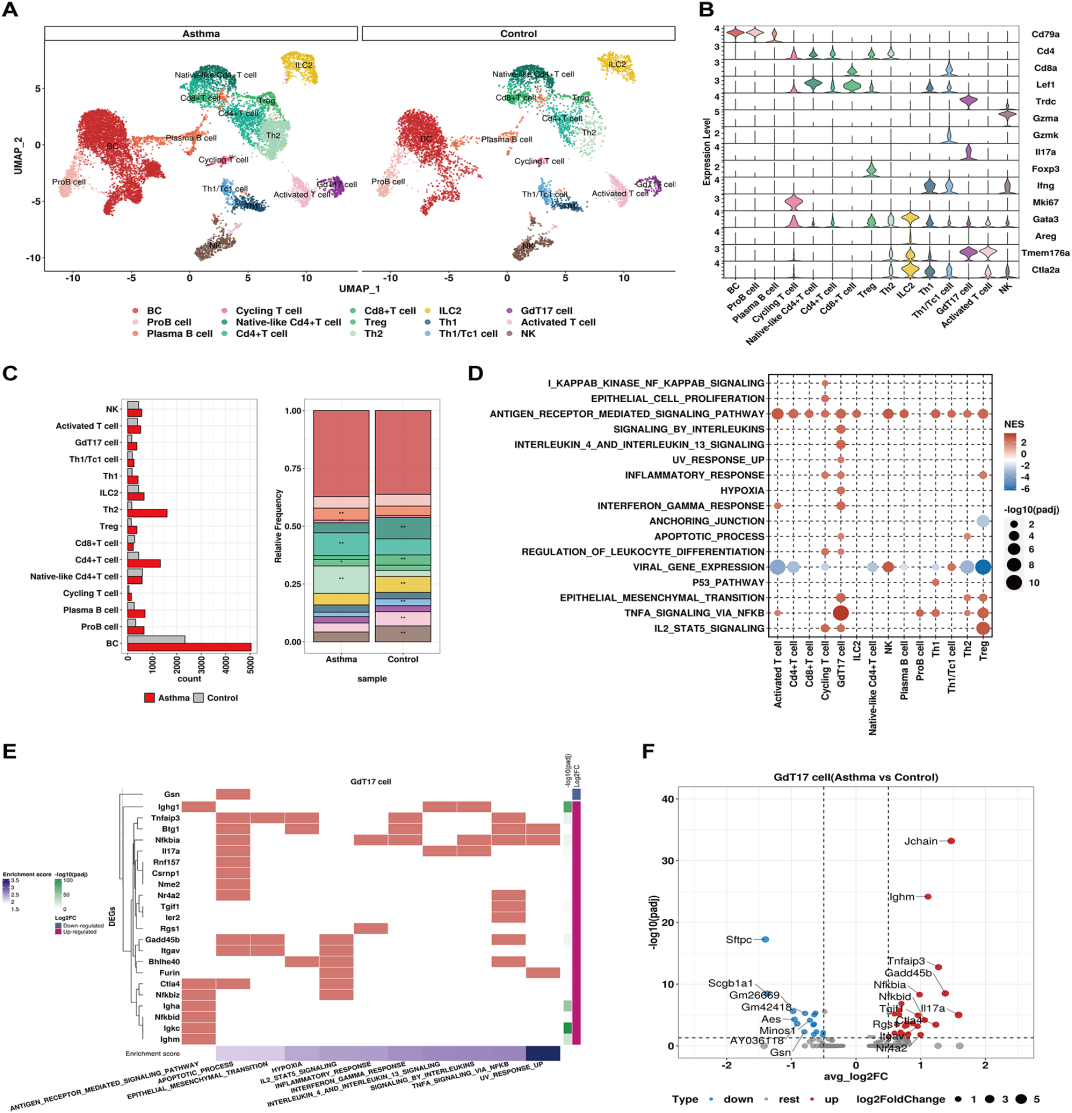
Characterization of lymphoid population in NC and AS groups. (**A**). A total of 15 clusters of lymphoid cells were identified. Each cell cluster was colored as indicated by the legend. (**B**) Stacked violin plots to visualize the expression level of signature gene markers in each cell population. (**C**) Left: the cell number of each cell cluster in NC and AS group; Right: representation of the relative cell proportion of each cell cluster, Fisher’s exact test computed significance of enrichment and highlighted with asterisks: *P < 0.05; **FDR < 0.05. (**D**) GSEA analysis identified significantly enriched signaling pathways in selected lymphoid cell clusters. All terms were significantly enriched (adjusted p-value < 0.05), dot size denoted -log10 (adjusted p-value) and normalized enrichment scores were shown in color legend. (**E**) Heatmap showing the DEGs involved in selected signaling pathways in GdT17 cell. Log2FC and -log10 (adjusted p-value) of gene were shown in color legend, enrichment score of signaling pathway was sorted and displayed in the bottom annotation bar. (**F**) Volcano plot displaying comparison of gene expression between the NC and AS group in GdT17 cell cluster. Up-regulated DEGs in AS group were highlighted in red and blue denoted down-regulated DEGs. Representative DEGs were labeled. The dot size represented the absolute log2Foldchange of gene expression

The lymphoid population was an important signaling receiver in the cell communication network, which was clustered into fifteen distinct subpopulations based on the established cell markers. We distinguished three B cell clusters: BC, Pro-B cell and Plasma B cell based on the expression of CD79a, Ifi30 and Jchain (Fig. 4A-B, Fig. S3). The proportion of Plasma B cells among total lymphoid cells dramatically increased after exposure to HDM (Fig. 4C), in agreement with previous studies indicating the involvement of IgE-producing plasma B cells in Th2-mediated inflammation [34, 41]. T cells were further classified into 11 subpopulations. Importantly, our model effectively captured the features of allergic inflammation, especially Th2-mediated eosinophilic inflammation, with a notable increase in the proportion of Th2 cells among total lymphoid cells after exposure to HDM. In the cell-cell communication analysis, GdT17 cells were recognized as one of the major signaling receivers from fibroblasts and macrophages in the AS group. Interestingly, GSEA analysis revealed that GdT17 cells in the AS group displayed upregulations in a variety of signaling pathways relevant to inflammatory response. These pathways included IL4-IL13 signaling, hypoxia, interferon-gamma response, epithelial-mesenchymal transition (EMT), TNFA-NFKB, and IL2-STAT5 signaling (Fig. 4D). Notably, key genes like TNFAIP3, NFKBIA, IL17A, and ITGAV, involved in at least two of the aforementioned pathways, were significantly up-regulated in GdT17 cells of AS (Fig. 4E-F, Table. S3). These observations suggested a vital role of GdT17 cells in promoting inflammatory response in asthma. Furthermore, GdT17 cells in AS was identified as a primary cellular resource of IL17 chemokine in this study, implying their close association with asthma severity, as IL17 has been linked to neutrophil activation and severe asthma [42].

### Macrophages played central role in the regulation of neutrophil properties in the context of asthma

**Fig. 5:**
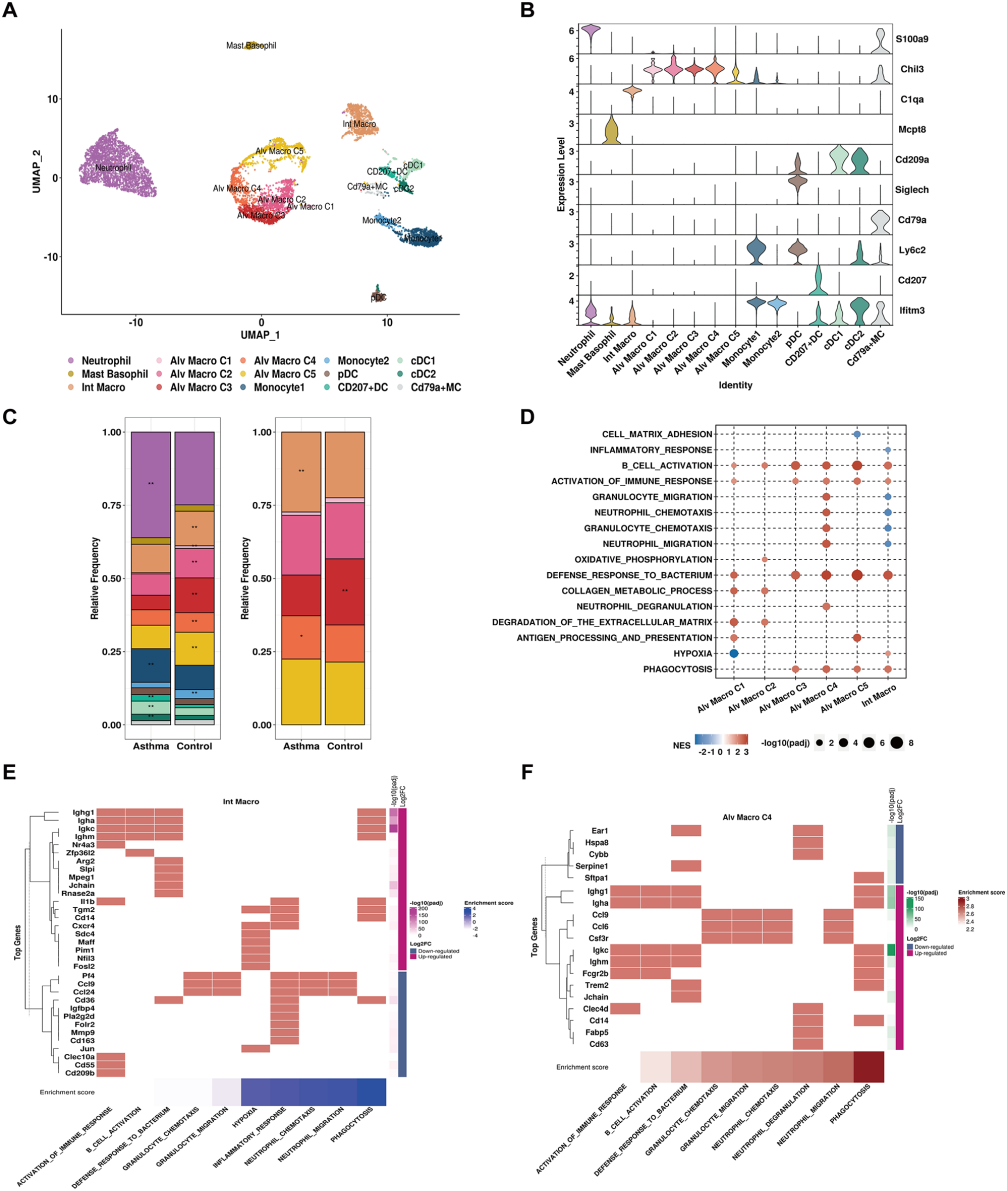
Characterization of myeloid population in NC and AS groups. (**A**) A total of 15 clusters of myeloid cells were identified. Each cell cluster was colored as indicated by the legend. (**B**) Stacked violin plots to visualize the expression level of signature gene markers in each cell population. (**C**) Left: the cell number of each cell cluster in NC and AS group; Right: representation of the relative cell proportion of each cell cluster, Fisher’s exact test computed significance of enrichment and highlighted with asterisks: *P < 0.05; **FDR < 0.05. (**D**) GSEA analysis identified significantly enriched signaling pathways in selected myeloid cell clusters. All terms were significantly enriched (adjusted p-value < 0.05), dot size denoted -log10 (adjusted p-value) and normalized enrichment scores (NES) were shown in color legend. Heatmap showing the DEGs involved in selected signaling pathways in (**E**) Int macro and (**F**) Alv macro C4. Log2FC and -log10 (adjusted p value) of gene were shown in color legend, enrichment score of signaling pathway was sorted and displayed in the bottom annotation bar

Considering the pivotal role of macrophages in cell communication network, we next investigate their roles and functions in asthma. Six distinct macrophage populations were identified based on transcriptomic profiles, referring to five subpopulations of Alv Macro and Int Macro based on the expression level of S100A9, CHIL3, C1QA, MCPT8, CD209A, SIGLECH, CD79A, LFITM3 and CD207 (Fig. 5A-B, Fig. S4A-B). Subpopulations of Alv Macro displayed distinct gene expression profiles relevant to pro-inflammatory macrophage (M1), anti-inflammatory macrophage (M2), and intermediate phenotypes. In addition to macrophages, neutrophils also played a crucial role in inflammatory responses, which represented the most significantly increased cell cluster in AS (Fisher’s exact test, FDR < 0.05, see Fig. 5C, Fig. S4C). Except for neutrophils, Int Macro and Alv Macro C4 were both remarkably increased in AS versus NC; hence, we next analyzed their potential functions related to the pathology of asthma. Intriguingly, Int Macro GSEA suggested upregulation of hypoxia and immune response with increased expression levels of CXCR4, SDC4 and FOSL2 in the HDM-induced model, alongside downregulations in the signaling pathways related to the chemotaxis and migration of neutrophils and granulocytes marked by downregulation of PF4, CCL9, and CCL24 (Fig. 5D-E, Table S4). In contrast to Int Macro, chemotaxis and migration of neutrophils and granulocytes as well as neutrophil degranulation were up-regulated in Alv Macro M4 of AS where these pathway-associated genes CCL9, CCL24 and CSF3R were all significantly up-regulated (Fig. 5F, Table S4). Macrophages and neutrophils were two major components in the regulation of the inflammatory microenvironment and they could even form a chronic inflammation phase associated with inevitable tissue remodeling [43]. Given that the frequent interactions between macrophages and neutrophils in cell communication network of asthma, these findings suggested that the infiltration of macrophages may contribute to airway inflammation in asthma by governing neutrophil properties.

### Heterogeneity of fibroblasts was associated with diverse pathological features of asthma

**Fig. 6:**
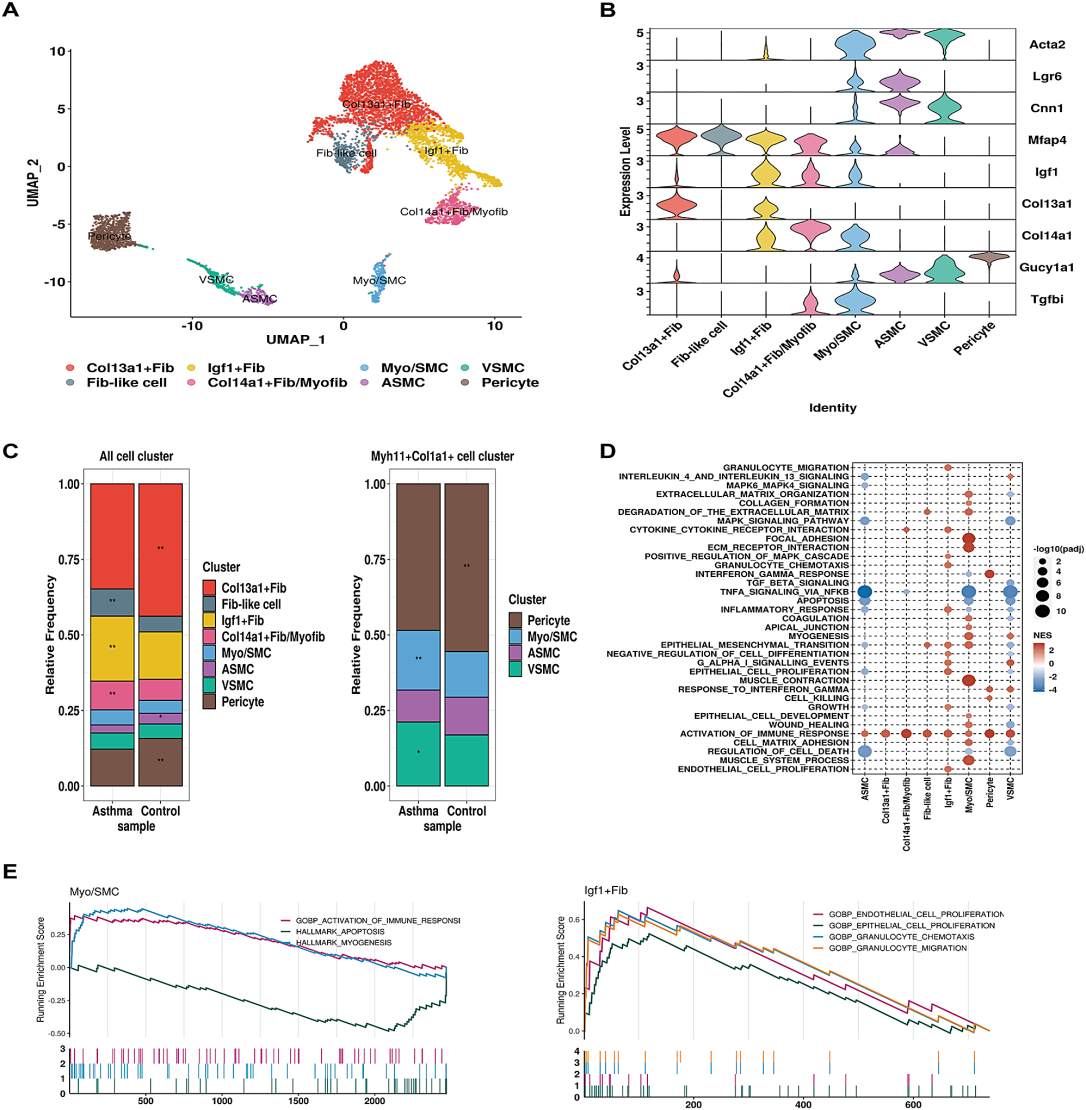
Characterization of stroma population in NC and AS groups. (**A**) A total of 8 clusters of stromal cells were identified. Each cell cluster was colored as indicated by the legend. (**B**) Stacked violin plots to visualize the expression level of signature gene markers in each cell population. (**C**) Left: the cell number of each cell cluster in NC and AS group; Right: representation of the relative cell proportion of each cell cluster, Fisher’s exact test computed significance of enrichment and highlighted with asterisks: *P < 0.05; **FDR < 0.05. (**D**) GSEA analysis identified significantly enriched signaling pathways in selected stromal cell clusters. All terms were significantly enriched (adjusted p-value < 0.05), dot size denoted -log10 (adjusted p-value) and normalized enrichment scores were shown in color legend. (**E**) GSEA plots depicting the enrichment of selected signaling pathways in Myo/SMC (left) and Igf1 + Fib (right) of AS group in comparison with NC group. Different signaling pathways were indicated by the color legend

As stroma was one of the major influencers within cell communication, we then got a deeper look into their roles in the pathogenesis of asthma. Eight cell clusters within the stroma were identified (Fig. 6A-B, Fig. S5A-B). Smooth muscle cell (SMC) was positive for contractile genes, MYH11, CNN1 and ACTA2, further subcategorized into airway smooth muscle cell (ASMC) and vascular smooth muscle cell (VSMC) based on the expression level of LGR6. Pericyte was characterized by high expression of GUCY1A1 and PDGFRB. Two distinct fibroblast populations were marked by COL13A1 and COL14A1 with a subpopulation of Col14a1 + fibroblast further defined as Col14a1 + Fib/Myofib marked by elevated expression levels of TGFBI and ACTA2 (Fig. 6A-B). Two additional fibroblast subsets were identified-one marked by highly expressed IGF1 (Igf1 + Fib) and another annotated as Fib-like cells with elevated expression of MFAP4. Myo/SMC appeared to represent an intermediate phenotype between myofibroblasts and SMC, characterized by ACTA2, MYH11, and TGFBI. Interestingly, the fraction of Igf1 + Fib and Col14a1 + Fib/Myo were increased whereas Col13a1 + Fib decreased in AS (Fig. 6C). Among all the MYH11 + COL1A1 + stromal cells, we observed a significant increase in the cell proportion of Myo/SMC and VSMC in AS compared to NC (Fisher’s exact test, FDR < 0.05), suggesting an abnormal increase of myofibroblast and VSMC associated with the pathology of asthma. Compared to NC, genes of Myo/SMC in AS were specifically enriched in myogenesis, wound healing, collagen formation, muscle contraction and degradation of ECM, whereas being depleted in apoptosis (Fig. 6D-E). Igf1 + Fib of AS was associated with inflammatory response, endothelial and epithelial cell proliferation as well as granulocyte migration and chemotaxis with associated gene expression changes as shown in Fig. S5C-D. These observations highlighted the association of heterogeneous fibroblasts with a variety of signaling pathways and biological functions related to airway inflammation and airway remodeling in asthma.

**Fig. 7:**
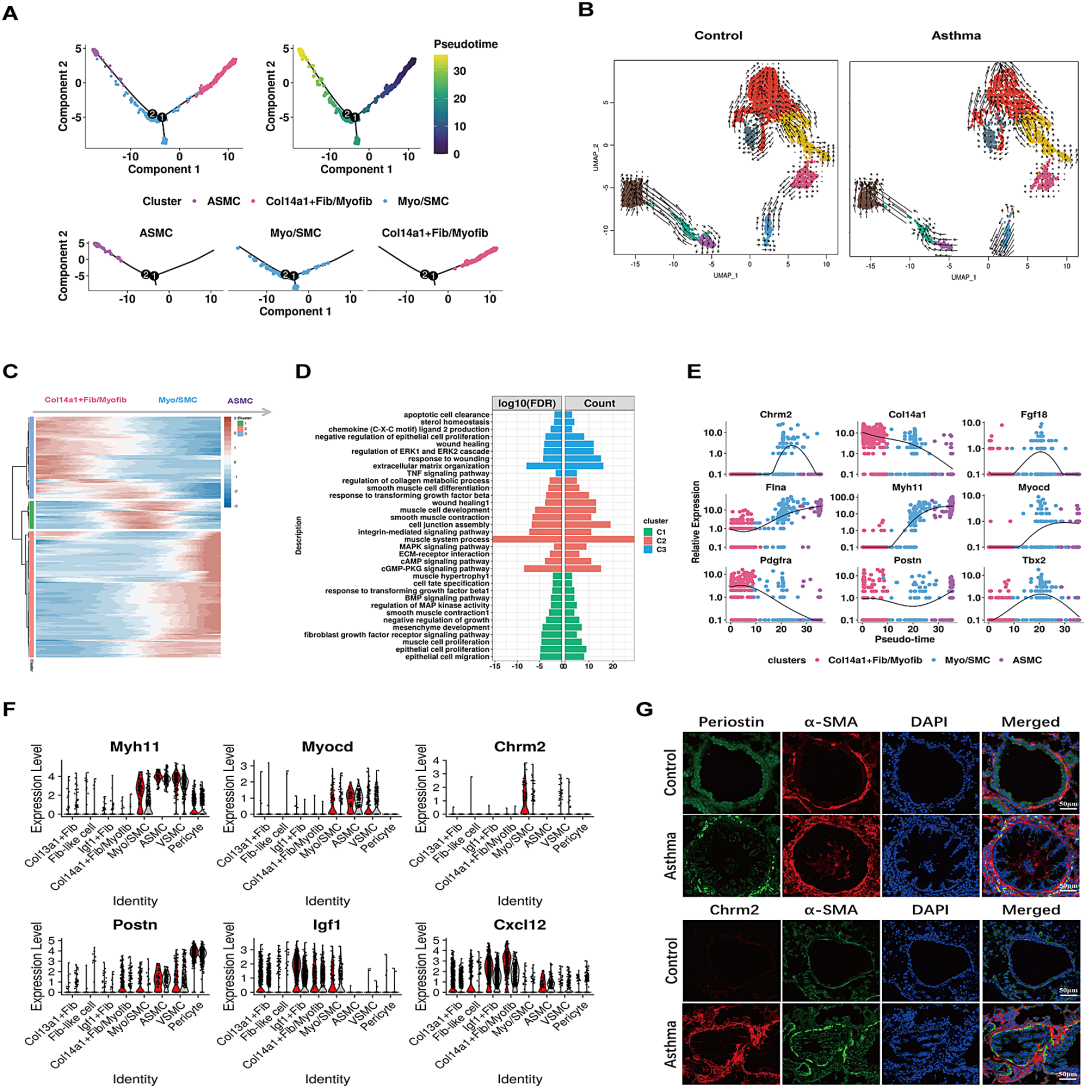
Pseudotime trajectory analysis assessing the cell fate of fibroblasts in asthma. (**A**) Pseudotime trajectory analysis to construct fibroblast to ASMC development trajectory. (**B**) RNA velocity analysis delineated dynamic changes in cell fate, projected onto a UMAP plot. Arrowheads indicated the predicted direction of cell development, with arrow size denoting the strength of predicted directionality. In the NC group (left), cells tend to move from ASMC to Col14a1 + Fib/Myofib, while in the AS group (right), there is a shift from Col14a1 + Fib/Myofib to ASMC. (**C**) Heatmap showing DEGs along the trajectory-based pseudotime from Col14a1 + Fib/Myofib through Myo/SMC to ASMC. The left-colored bars represented gene cluster C1 to C3. (**D**) Functional enrichment analysis revealing KEGG pathways and GO terms significantly associated with gene cluster C1 to C3, as depicted in the left figure; the two panels referred to the log10(FDR) and gene count ratio, respectively. (**E**) Dynamic changes in the expression level of selected DEGs along pseudotime. (**F**) Violin plots illustrating expression level of selected genes in NC (grey) and AS group (red). (**G**) Representative immunofluorescence image demonstrating the co-localization of top: POSTN and α-SMA (ACTA2) and bottom: CHRM2 and α-SMA in NC and AS groups

Monocle2 and RNA velocity analysis indicated a potential trans-differentiation of myofibroblasts into ASMC, with Myo/SMC serving as an intermediate cell state (Fig. 7A-B). Monocle tracked the changes of gene expression over pseudotime along Col14a1 + Fib/Myofib, Myo/SMC to ASMC, revealing three gene clusters C1-C3 (Fig. 7C). GO enrichment analysis highlighted C3 genes (e.g., COL14A1, PDGFRA, MMP3, MMP14, CCL8 and CXCL12) at the inception of the trajectory, enriched in wound healing, ECM and TNF signaling pathway, while C2 genes (e.g., FLNA, TAGLN, MYOCD, MYH11, ACTA2) at the end of the trajectory were associated with smooth muscle cell differentiation and contraction (Fig. 7D-E, Table S5). CXCL12, a chemokine pivotal for immune cell homing, migration, and survival through CXCR4 binding, was highly expressed by the majority of stromal cells. In our cell communication analysis, CXCL12-CXCR4 emerged as the primary ligand-receptor pair orchestrating interactions between fibroblast subpopulations and immune cells, indicating its significant role in immune activation and inflammatory response in asthma. Furthermore, C1 genes (e.g., CHRM2, TBX2, HHIP, BMP5, FGF1, ACTC1) from the intermediate state were correlated with cell fate specification and response to both TGFB and BMP signaling pathways (Fig. 7D-E). Interestingly, most contractile genes such as MYH11, TAGLN, CNN1, ACTA2 as well as regulatory genes MYOCD, IGF1 and CXCL12 were all up-regulated in the Myo/SMC of AS group (Fig. 7F, Table S6). Additionally, POSTN, serves as a biomarker for airway inflammatory response and airway remodeling, was highly expressed in Col14a1 + Fib/Myofib, Myo/SMC, ASMC and pericyte in AS group. Besides, Myo/SMC in AS exhibited high expression in CHRM2, a gene determining contractile tension in smooth muscle, despite not statistically significant. These finding were further validated in mouse lung tissue sections, confirming increased expression of POSTN and CHRM2 in ACTA2 + cells in AS compared to NC (Fig. 7G). Myofibroblasts tend to migrate to airway smooth muscle bundles, and trans-differentiate into smooth muscle-like cells with up-regulated CHRM2, potentially contributing to excessive contractile responses and increased airway responsiveness. Collectively, the diversity within the fibroblast populations emerged as a central hub, influencing not only airway inflammation and remodeling but also contributing to ASM contraction in asthma.

### Macrophage-fibroblast interaction as key contributor to airway inflammation and remodeling in asthma

**Fig. 8:**
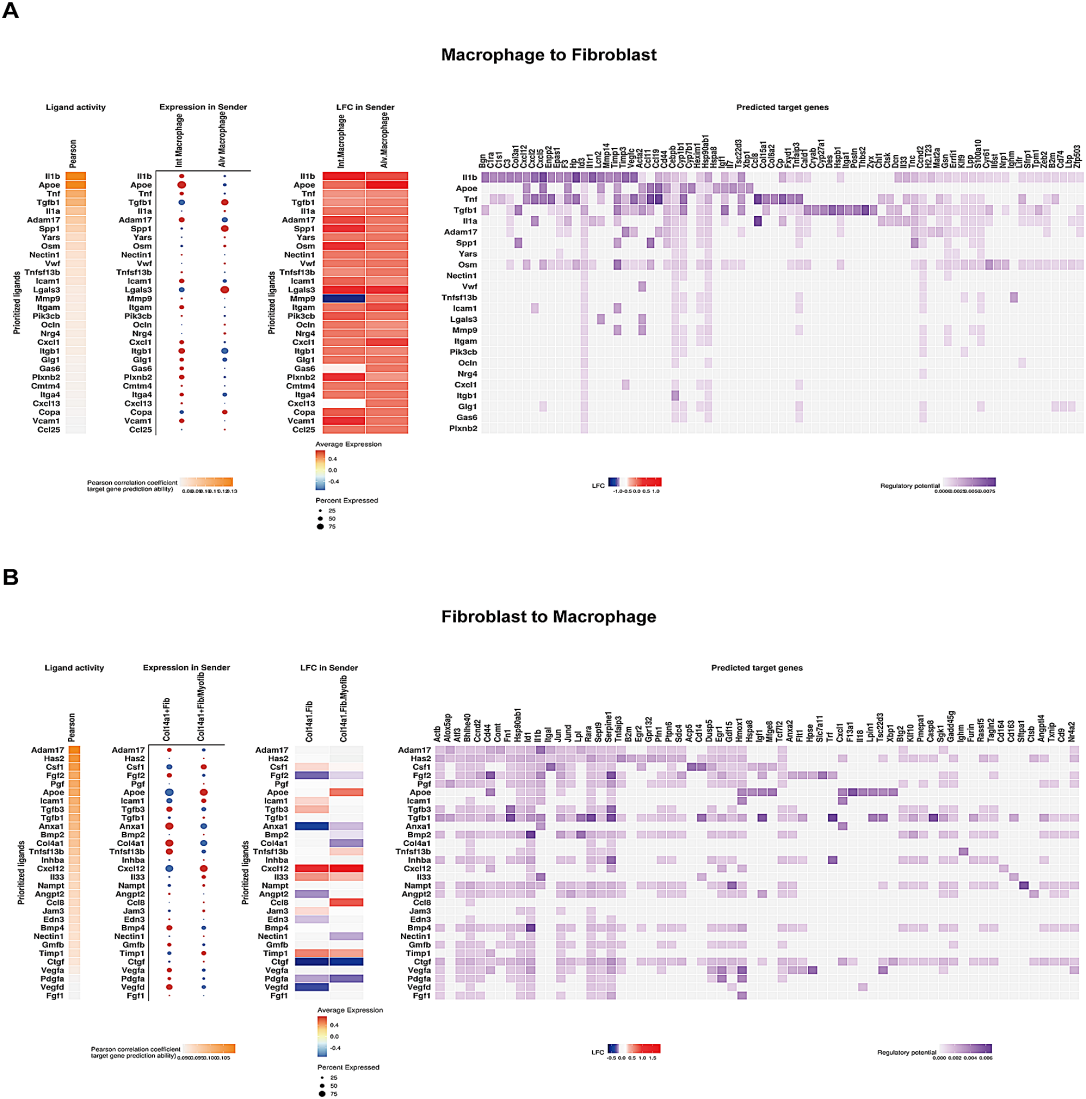
Deciphering the cell interactions between macrophage and fibroblast subpopulations associated with airway inflammation and remodeling. The top30 ligand activity and the expression level of these ligands in sender cells (**A**) int macrophage and alv macrophage; and (**B**) Col14a1 + Fib and Col14a1 + Fib/Myofib) to their respective receiver cells were presented. Log2FoldChange of these ligand expression values, comparing AS to NC group, and the predicted target genes in receiver cells were identified by NicheNet analysis

Given that macrophage and fibroblast populations as key modulators in the cell interaction network of AS, we aimed to investigate the contribution of interactions between macrophage and fibroblast subpopulations to the pathogenesis of asthma. Two subpopulations of macrophage (Int Macro and Alv Macro) interacted with Col14a1 + Fib/Myofib and Col14a11 + Fib by IL1B, TGFB1 and SPP1 signaling pathways (Fig. 8A). In AS, the target genes of these subpopulation of fibroblast were enriched in the pathways relevant to epithelial cell proliferation and inflammatory response, including neutrophil chemotaxis and migration, where the target genes POSTN, IGF1, CCL11, CXCL5 and CXCL12 were significantly up-regulated in AS (Fig. S6). On the other hand, fibroblasts interacted with Int Macro and Alv Macro through various ligands, including CCL8, CXCL12 and APOE, which were up-regulated in Col14a1 + Fib/Myofib of AS (Fig. 8B, Table S6). Target genes of Int Macro were highly relevant to wound healing, muscle cell proliferation, interleukin-1 beta production and lymphocyte differentiation (Fig. S7A-B). Specific to asthma, macrophages engaged with fibroblasts via the IL1B signaling pathway, and IL1B was identified as a target gene of macrophages regulated by fibroblasts, which exhibited particularly significant up-regulation in the Int Macro (Fig. S7C). This suggested the existence of a positive feedback loop in cell-cell communication between fibroblasts and macrophages, particularly the Int Macro. Collectively, our findings demonstrated that interactions between macrophages (Int Macro and Alv Macro) and fibroblasts (Col14a1 + Fib, Col14a1 + Fib/Myofib) played a pivotal role in both airway inflammation and remodeling in asthma.

## Discussion

Increasing evidence has demonstrated that cell-cell communications play important roles in the development of human diseases [44]. Exploring the cell-cell interactions implicated in cellular dysfunctions associated with asthmatic pathogenesis may contribute to identifying novel biomarkers and therapeutic targets. In this study, we established a 16-week mouse model with HDM intranasal administration to induce allergic airway inflammation and airway remodeling. We conducted a comprehensive analysis of cellular composition, expression profiles, and cell communication networks in both normal and asthmatic lungs through scRNA-seq. Our findings provide more insights into the pathogenesis of airway inflammation and airway remodeling, shedding light on key immune and structural cell populations and determining the cell-cell communication patterns that contribute to these processes.

Previous studies have demonstrated that exposure to HDM induces allergic asthma in mice, marked by remarkable increase in immune cell counts, including eosinophils, neutrophils, lymphocytes, and macrophages in BALF, as well as increased airway hyperresponsiveness [17, 45]; consistently, we observed these asthma symptoms in our mouse model, indicating the successful establishment of an asthmatic phenotype. Considering time and cost, 4-week model of asthma with typical airway inflammation and hyperresponsiveness was normally established. However, induction of airway remodeling requires longer duration of allergen exposure, typically 8–12 weeks. Nonetheless, current research found that airway remodeling features in 4 and 8 weeks are comparable [17]. In comparison to the mouse model of OVA-induced acute asthma, a chronic (12-week) model revealed an increase in airway smooth muscle mass, although not statistically significant [46]. To unravel the pathological mechanisms underlying airway inflammation and airway remodeling comprehensively, we considered establishing an asthma model with extensive structural remodeling features, particularly a significant increase in airway smooth muscle mass. This was achieved by prolonging HDM exposure to 16 weeks, ensuring the sustained development of airway remodeling. Histological analysis, including H&E, PAS and MASSON staining analysis, respectively revealed significant inflammatory cell infiltration, mucus hyperproduction, goblet cell hyperplasia and increased collagen deposition in our mouse model. In addition, measurements of airway wall thickness [47] demonstrated a significant thickening of the airway wall in AS compared to NC. Moreover, immunofluorescent analysis further confirmed the increased airway wall thickness and revealed elevated expression of α-SMA, a biomarker for both ASMC and myofibroblast [48, 49]. Furthermore, our HDM-induced allergic asthma model exhibited a mixed granulocytic inflammation with prominent neutrophilia, aligning with previous reports [50]. Overall, these observations supported the successful establishment of a 16-week mouse model of HDM-induced asthma, characterized by significant airway inflammation, remodeling, and hyperresponsiveness.

We employed scRNA-seq analysis on the entire lungs of mice to delineate key cell clusters and their functions in asthma. Our analysis identified 34 distinct cell clusters, encompassing myeloid, lymphoid, epithelial, stromal, and mesothelial cell clusters, which closely aligned with recently published data [28, 34, 51, 52]. In agreement with previous studies [51, 53], Th2 cells were markedly increased in asthmatic model, indicating robust allergic airway inflammation induced by HDM sensitization and repetitive challenges. Eosinophil infiltration was related to the development of asthma exacerbation and even airway remodeling in severe asthma [54, 55], although we observed a significant increase of eosinophil in BALF, they were not detected by scRNA-seq analysis due to the technological limitation [52] and thus hindering our understanding of its cellular contributions to disease. Conversely, the substantial increase in neutrophils identified by both BALF and scRNA-seq analysis suggested that our 16-week model potentially represents severe chronic asthma as airway neutrophilia has been associated with asthma exacerbation and severity [56]. Notably, GdT17 cells identified in our model of asthma were enriched for TRDC and pro-inflammatory cytokine IL-17 A, positioning them as major cellular sources of IL-17 [57, 58]. Specifically, GSEA analysis revealed GdT17 cells in asthma model were associated with various signaling pathways, including IL4/IL13 signaling, IFN-γ response and inflammatory response. IFN-γ, a key driver of airway neutrophil recruitment and overall lung inflammation [59] along with IL4 and IL13, pivotal Type 2 cytokines, underscored the complex regulatory network underlying allergic inflammation in asthma [60]. IL17 cytokines have been implicated in the asthmatic pathogenesis, severity as well as corticosteroid (CS) resistance, particularly in neutrophilic asthma [61, 62], and were significantly up-regulated in GdT17 cells of the asthma model [63, 64]. These findings highlighted the pivotal role of GdT17 cells in amplifying inflammation, potentially shaping the endotypes of asthma, and warrant further investigation and clinical studies for therapeutic targeting.

Moreover, macrophages, identified as another highly activated cell cluster, played multifaceted regulatory functions in asthma. Int Macro and Alv Macro exhibited distinctive expression profiles and functional characteristics. The increased proportion of Int Macro in the asthma model, characterized by down-regulation of genes related to neutrophil and granulocyte migration, chemotaxis, and inflammatory response (e.g., CCL9, CCL24, PF4) and upregulation of genes related activation of immune response and bacterial defense (e.g., IL1B and SLPI), suggested a potential protective role against inflammation. These observations highlighted their unique immunoregulatory activities as described in previous studies [65, 66]. Alv Macro, known to be either M1 or M2, were clustered into five subsets distinguished by specific marker genes. Pro-inflammatory hallmark genes CXCL15 and MALAT1 were uniquely overexpressed in Alv Macro C5, indicating a potential role in promoting inflammation. In contrast, four subsets (Alv Macro C1, C2, C3 and C4) likely represented M2 macrophage characterized by highly expressed anti-inflammatory genes MMP12, CCL6 and SPP1. Despite Alv Macro C1 representing a small subset, cell-cycling genes such as TOP2A and MKI67 were uniquely highly expressed, suggesting a role in mediating the biological process from injury to tissue regeneration, such as wound healing [67]. GSEA analysis revealed that Alv Macro C4 was significantly enriched in genes (e.g., CCL6, CCL9, and CSF3R) associated with neutrophil migration, chemotaxis, and degranulation, implying a predominant role in modulating neutrophil activation. Macrophage activities, including polarization, autophagy, and phagocytosis, have been heavily associated with asthma development and severity [68, 69]. Macrophages exhibit dual roles, capable of either promoting or inhibiting airway inflammation and remodeling, with their phenotype switching from M1 to M2. The complexity of their dual roles and plasticity necessitates further efforts for a comprehensive understanding.

Active fibroblasts, particularly myofibroblasts, play a crucial role in airway remodeling and have been extensively studied in conditions such as COPD and fibrosis; however, their implication in asthma remains less understood [70]. Our study identified four specific clusters of fibroblasts characterized by COL13A1, COL14A1, ACTA2 and IGF1. Notably, we observed a significant increase in the proportion of IGF1 + Fib and Col14a1 + Fib/Myofib in the asthmatic stromal cell subclusters. Enrichment analysis revealed that the former was involved in a variety of inflammatory regulation pathways, while the latter was more correlated with wound healing and ECM. Two trajectory analysis tools, Monocle2 and RNA velocity, independently identified that myofibroblasts potentially trans-differentiate into ASMC contributing to increased airway smooth mass in asthma, as speculated in previous studies [71, 72]. However, myofibroblasts can be derived from different cell types, thus, further experimental validations are invited to make it more clear about all their origins and destinations in asthma. We observed a significant upregulation of the chemokine CXCL12 in all subpopulations of fibroblast in the asthma model. CXCR4, which governs immune cell recruitment, was widely expressed in immune cells. Accumulating evidence also suggests that CXCL12/CXCR4 signaling plays an important role in shaping the microenvironment, mediating allergic airway inflammation (such as retaining neutrophils at inflammatory sites) and promoting airway remodeling (e.g., induction of EMT) in asthma [35, 73]. This indicated that CXCL12/CXCR4 axis serves as a pivotal signaling pathway for fibroblast communication with immune cells that contributes to the pathogenesis of asthma. Intriguingly, Myo/SMC, identified as an intermediate state between fibroblasts and ASMC, exhibited high cell plasticity and overexpression of contractile genes CNN1, MYH11, and ACTA2, along with T2 inflammation-relevant genes POSTN and the CHRM2-regulatory hallmark element of contractile protein genes. This observation was validated by immunofluorescence analysis. Moreover, functional analysis showed that asthmatic Myo/SMC was particularly associated with various signaling pathways relevant to airway remodeling. Collectively, these findings underscore the importance of determining fibroblast cell fate as a novel breakthrough in developing asthmatic treatment strategies. Targeting fibroblast dynamics holds potential to improve asthma control and inhibit severity by attenuating or reversing airway remodeling.

To systematically analyze the cell-cell communication in asthma, our study highlighted strengthened interactions between stromal-myeloid and stromal-lymphoid cells, which was consistent with prior research [34, 39]. Notably, fibroblasts emerged as key modulator in both airway inflammation and remodeling, particularly in their interactions with GdT17 cells and ILC2. Besides, we observed a decrease in communication between epithelial and immune cells, possibly due to the severe epithelial damage in our asthma model. Our study revealed that fibroblasts and macrophages, especially Int Macro, were major influencers in the overall cell communication network. They engaged in paracrine/autocrine signaling, dominated by up-regulated signaling pathways such as CXCL12, POSTN, IL1B, CCL8, and IGF1. Specifically, the interaction between Int Macro and Col14a1 + Fib/myofib dominated the fibroblast-macrophage interactions. NicheNet analysis revealed that IL1B, overexpressed in asthmatic Int Macro, was a prioritized ligand for macrophages to interact with fibroblast subpopulations whose prominent target genes (e.g., CCL11, IGF1 and CXCL12) involved in immune response and regulation of neutrophil functions were significantly up-regulated in asthma. Surprisingly, we observed that IL1B, involved in wound healing and smooth muscle cell proliferation in Int Macro, was a downstream target gene regulated by fibroblast subpopulations. This suggests a novel feedback loop between macrophages and fibroblasts specific to asthma, with macrophages promoting inflammatory regulation in fibroblasts, and being regulated by fibroblasts to mediate airway remodeling. Additionally, cell-cell communication analysis revealed that neutrophil was the central signaling receiver from both macrophages and fibroblast subpopulations via IGF1-IGF1R and CXCL12-CXCR4 (Fig. S8). Given that IGF1 could delay apoptosis of neutrophils while CXCL12/CXCR4 was key retention and migration signal for neutrophil activities [74, 75], fibroblast and macrophage cell circuits potentially exacerbate inappropriate retention and anti-apoptosis of neutrophils at inflammatory sites, acting as a major driver of the excessive damage characteristics in asthma. The precise mechanisms underlying these interactions require further investigation. Nevertheless, it can be concluded that macrophage-fibroblast communication likely shapes the inflammatory microenvironment and promotes the connections between airway inflammation and remodeling.

Despite these insights, our study has limitations, including a relatively small sample size for scRNA-seq analysis (3 mice for both the model and control). We observed individual variations in total cell number among the murine of model. Secondly, it would be better to compare our model to the 4-week and 8-week asthma model, which would provide supportive evidence to verify the 16-week model of asthma is characterized by substantial structural remodeling close to asthma, even representing severe asthma. Moreover, the role of eosinophils, not detectable in mouse single-cell RNA sequencing, requires additional investigation such as cell sorting. Multi-timepoint scRNA-seq experiments would evaluate dynamic changes in gene profiles and communication signaling during the inception and progression of airway remodeling. Our study highlights the potentially central role of int macrophage-fibroblast cell circuits in modulating the development of airway inflammation and remodeling. We encourage further clinical trials, in vitro studies (e.g., organoids), as well as in vivo investigations, to refine our understanding of the causality between airway inflammation and remodeling. Lastly, while the 16-week model demands considerable time and cost, improvement of the asthma model at a low cost to replicate typical features of human asthma will facilitate the identification of underlying regulatory mechanisms and potential interventions to attenuate or even reverse airway remodeling.

## Conclusion

In summary, we established a 16-week murine model of HDM-induced allergic asthma, characterized by airway inflammation, airway remodeling, and airway hyperresponsiveness. Using scRNA-seq, our analysis provided a comprehensive understanding of cellular and functional heterogeneity in the asthma model. We identified key components driving airway inflammation and remodeling in asthma, shedding light on the molecular mechanisms underlying both structural changes and inflammatory responses. Our findings refined the knowledge of the crucial roles of GdT17 cells, macrophages and fibroblasts in contributing to asthma pathogenesis, complementing prior studies. A systematic investigation of cell-cell communication highlighted that stromal-immune cell interactions are of great importance in propelling the progression of airway inflammation and airway remodeling in asthma. This study enhances our insights into the precision treatment strategies targeting both airway inflammation and remodeling, particularly the blockade of inappropriate macrophage-fibroblast interactions via specific ligand-receptor pairs.

### Electronic supplementary material

Below is the link to the electronic supplementary material.

Supplementary Material 1

Supplementary Material 2

Supplementary Material 3

Supplementary Material 4

Supplementary Material 5

Supplementary Material 6

Supplementary Material 7

Supplementary Material 8

Supplementary Material 9

Supplementary Material 10

## Data Availability

No datasets were generated or analysed during the current study.

## Abbreviations

ICS: Inhaled corticosteroids
ASM: airway smooth muscle
HDM: House dust mite
scRNA-seq: Single-cell RNA sequencing
BALF: Bronchoalveolar lavage fluid
sRaw: Specific airway resistance
H&E: Hematoxylin-Eosin
PAS: Periodic Acid-Schiff
UMI: unique molecular identifier
UMAP: Uniform Manifold Approximation and Projection
DEGs: Differential expression genes
GSEA: Gene Set Enrichment Analysis
Int Macro: Interstitial macrophages
Alv Macro: Alveolar macrophages
NC: normal control group
AS: asthma group
Fib: Fibroblast
Myofib: Myofibroblast
EMT: Epithelial-mesenchymal transition
M1: Pro-inflammatory macrophage
M2: Anti-inflammatory macrophage
SMC: Smooth muscle cell
ASMC: Airway smooth muscle cell
VSMC: Vascular smooth muscle cell
