# Supplementary figures and images for: Single-cell analysis reveals alterations in cellular composition and cell-cell communication associated with airway inflammation and remodeling in asthma
Source: Respir Res. 2024 Feb 5;25:76. doi: 10.1186/s12931-024-02706-4 (PMC10845530; doi:10.1186/s12931-024-02706-4)

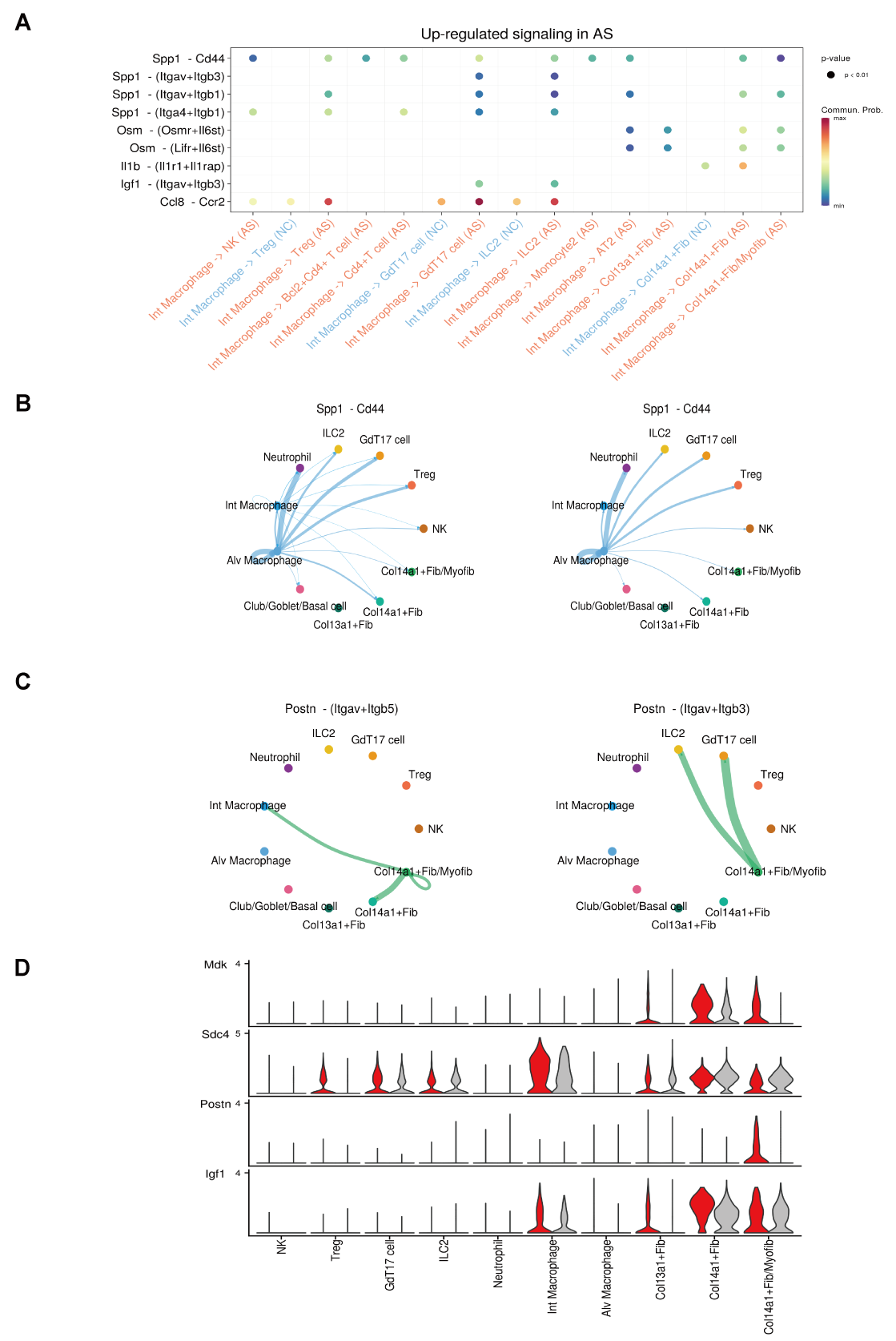

Supplement: Supplementary file 1 — Supplementary Material 1 [file 12931_2024_2706_MOESM1_ESM.tif]

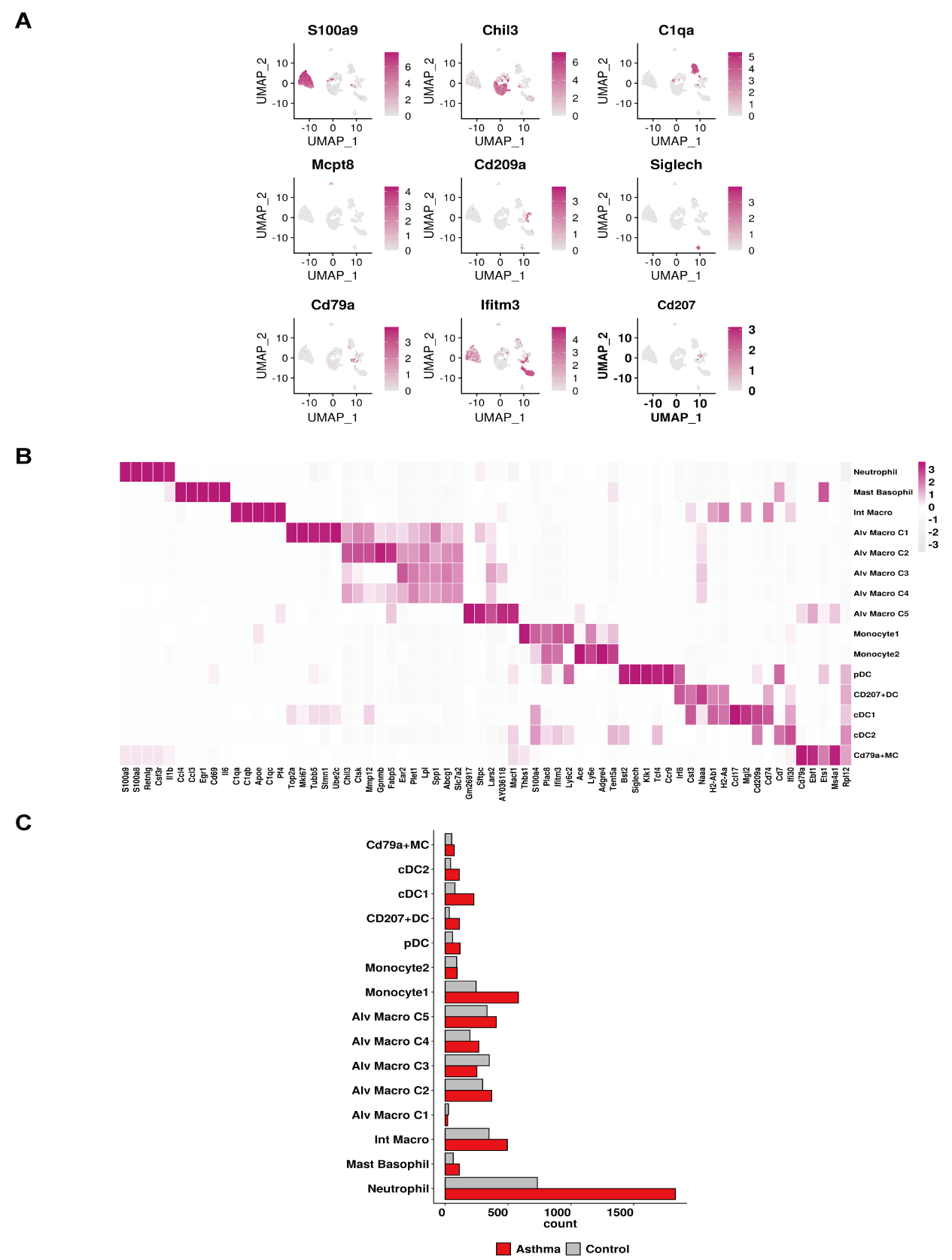

Supplement: Supplementary file 2 — Supplementary Material 2 [file 12931_2024_2706_MOESM2_ESM.tif]

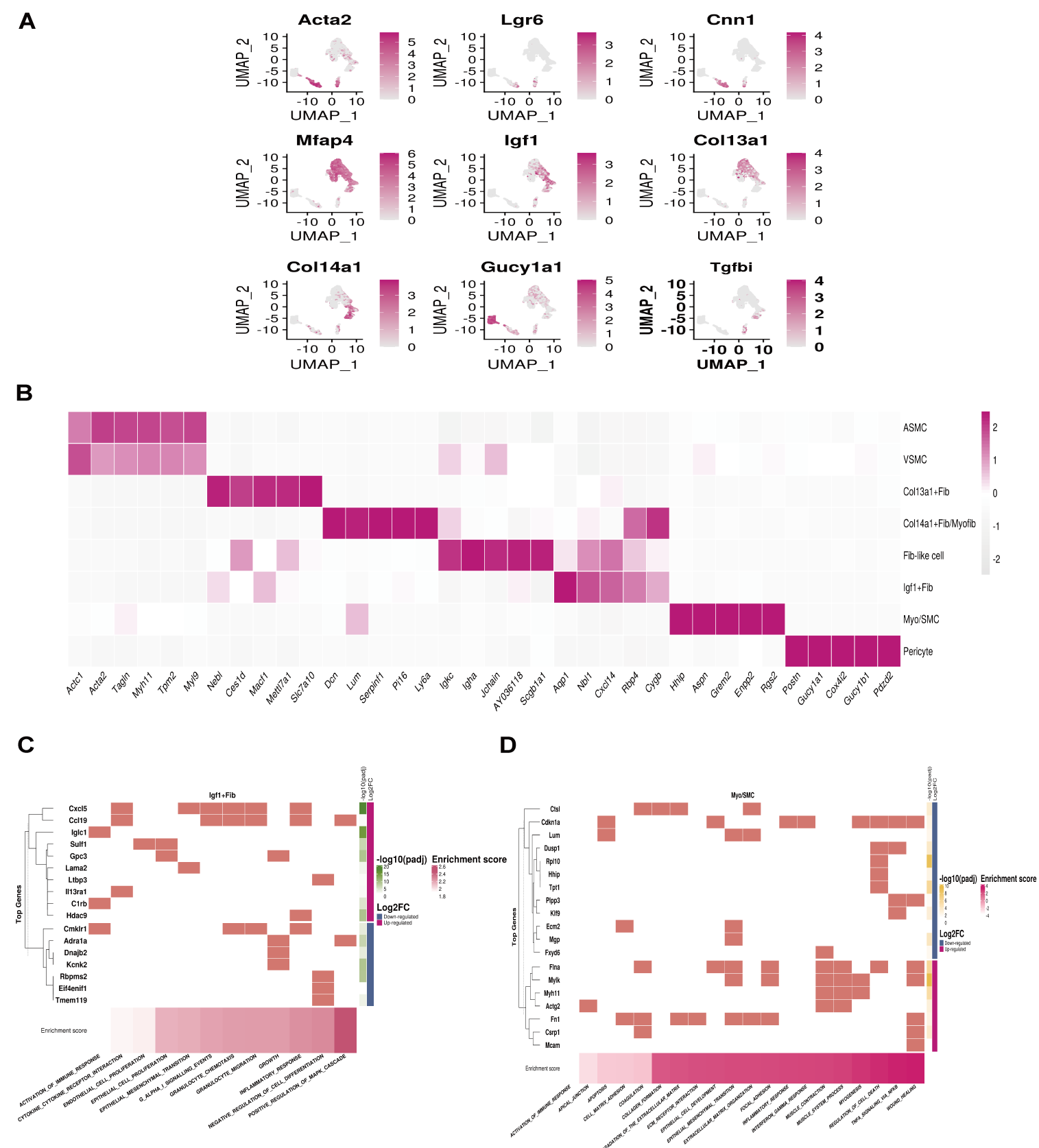

Supplement: Supplementary file 3 — Supplementary Material 3 [file 12931_2024_2706_MOESM3_ESM.tif]

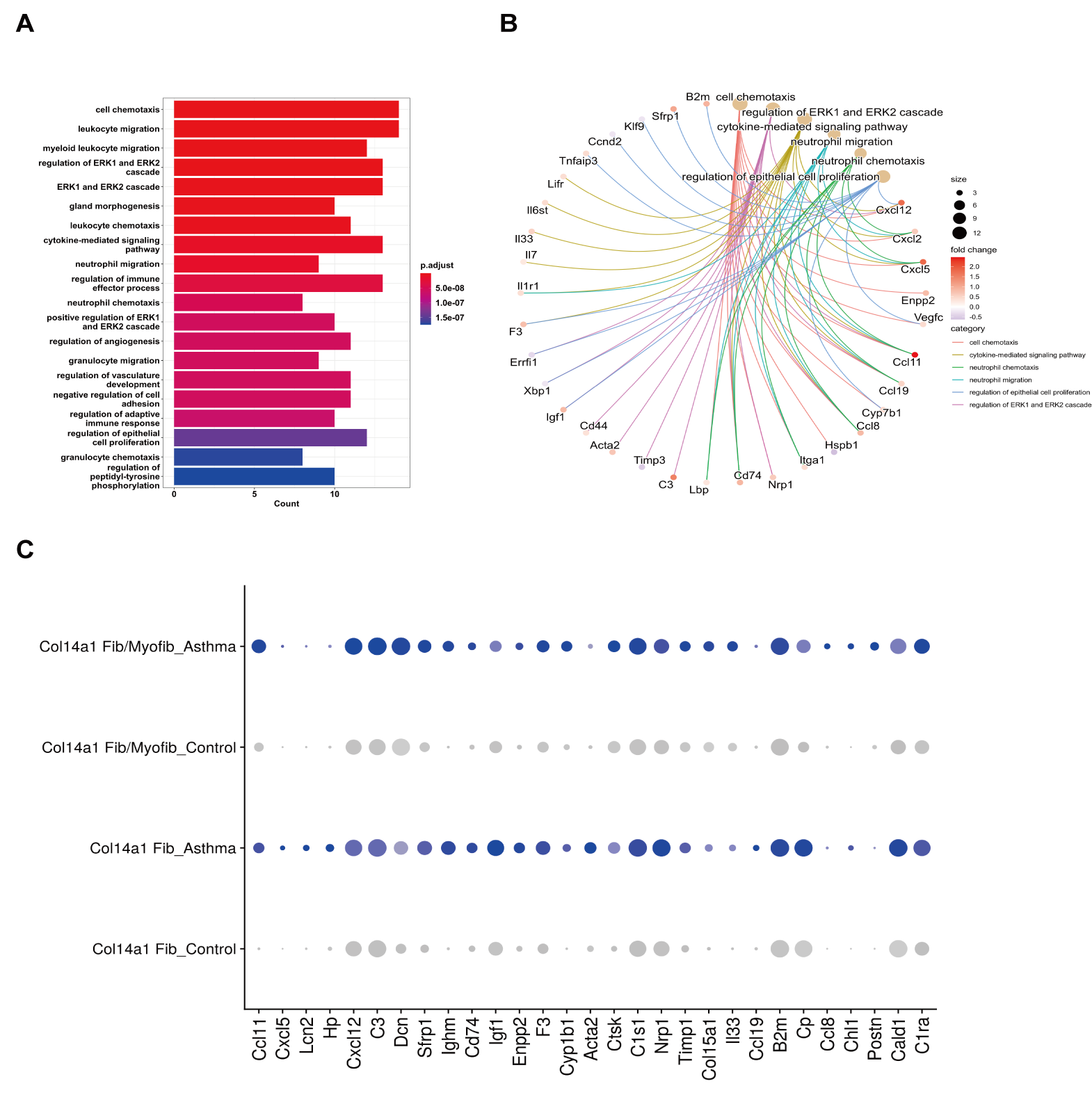

Supplement: Supplementary file 4 — Supplementary Material 4 [file 12931_2024_2706_MOESM4_ESM.tif]

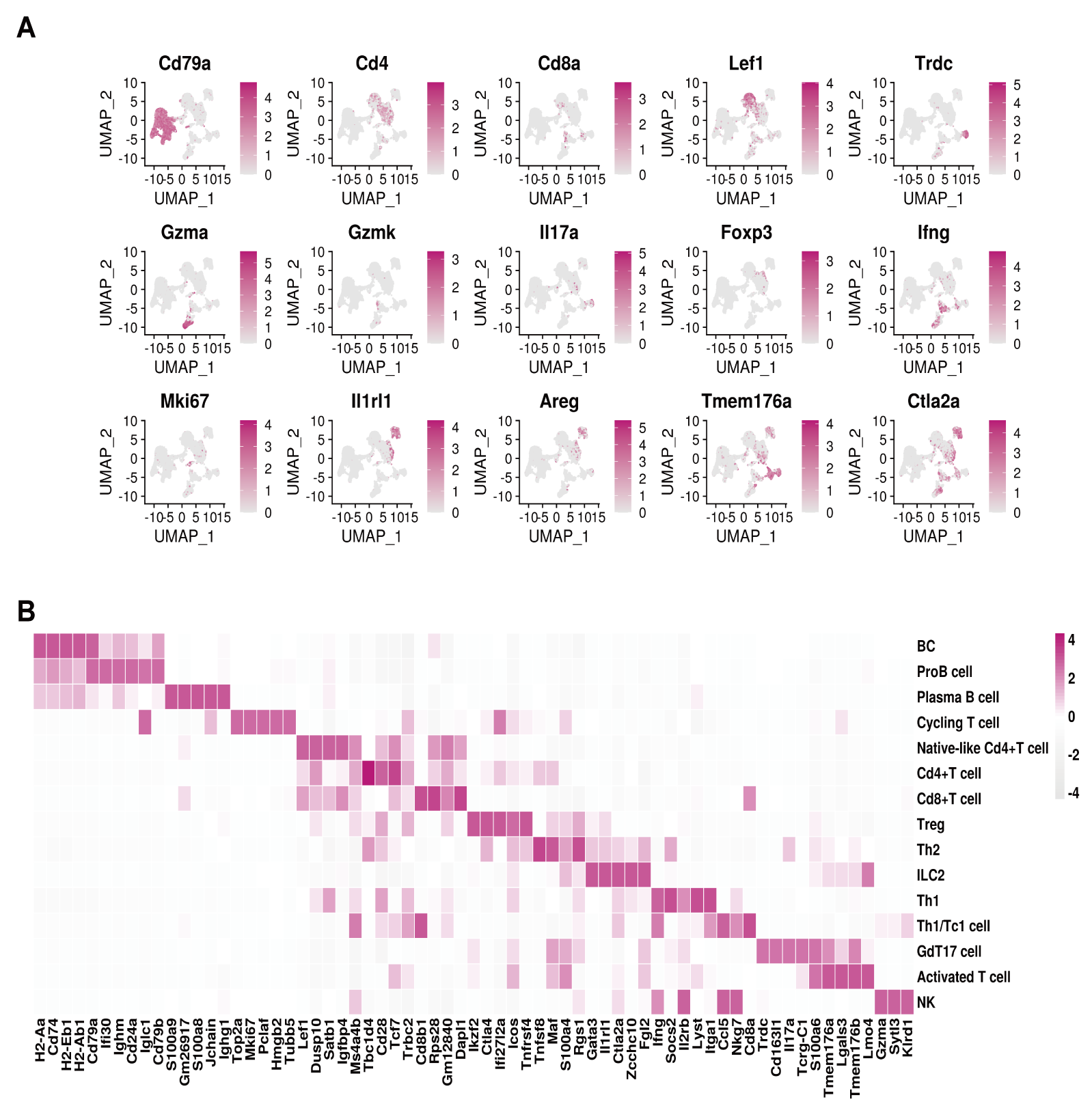

Supplement: Supplementary file 5 — Supplementary Material 5 [file 12931_2024_2706_MOESM5_ESM.tif]

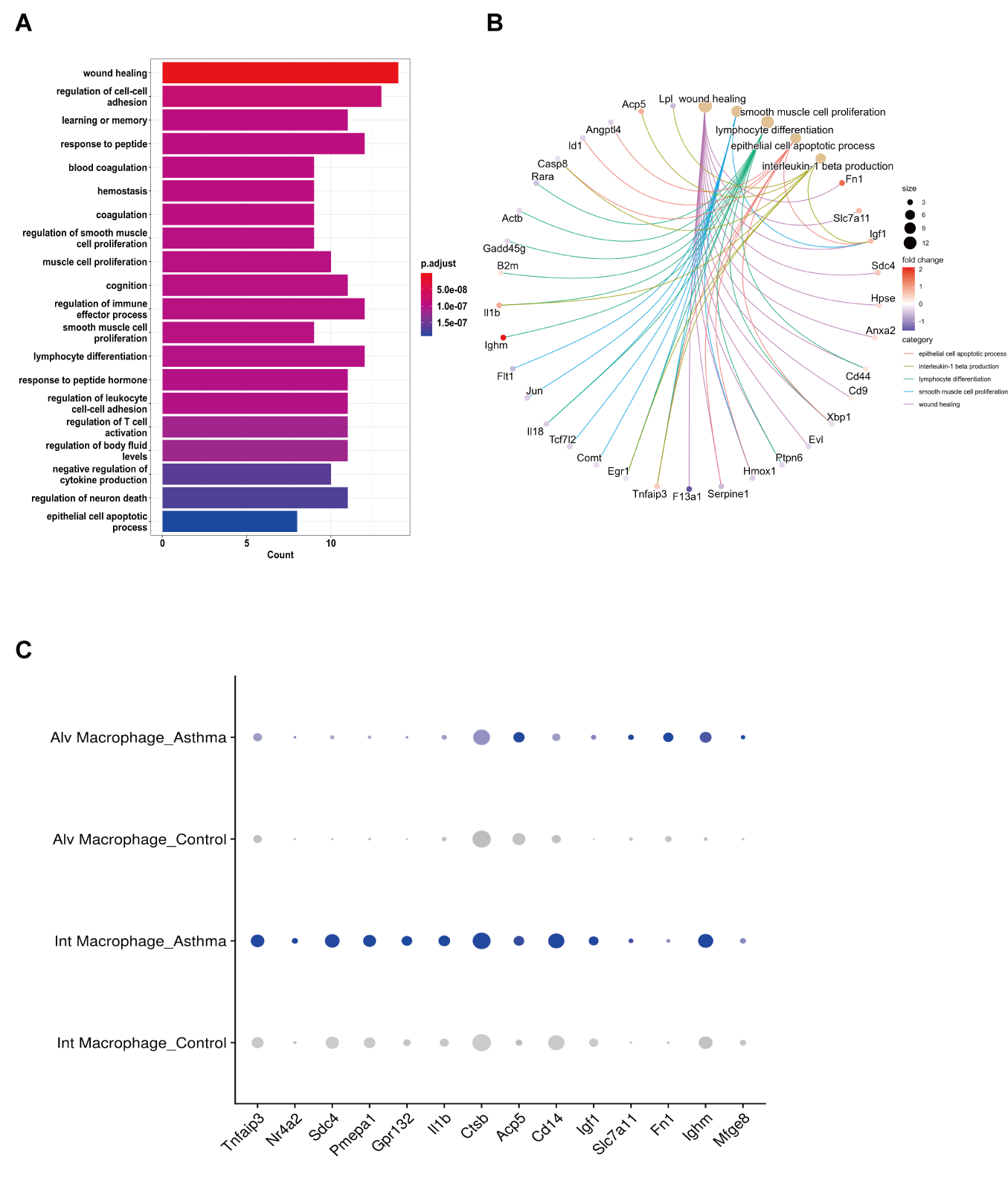

Supplement: Supplementary file 6 — Supplementary Material 6 [file 12931_2024_2706_MOESM6_ESM.tif]

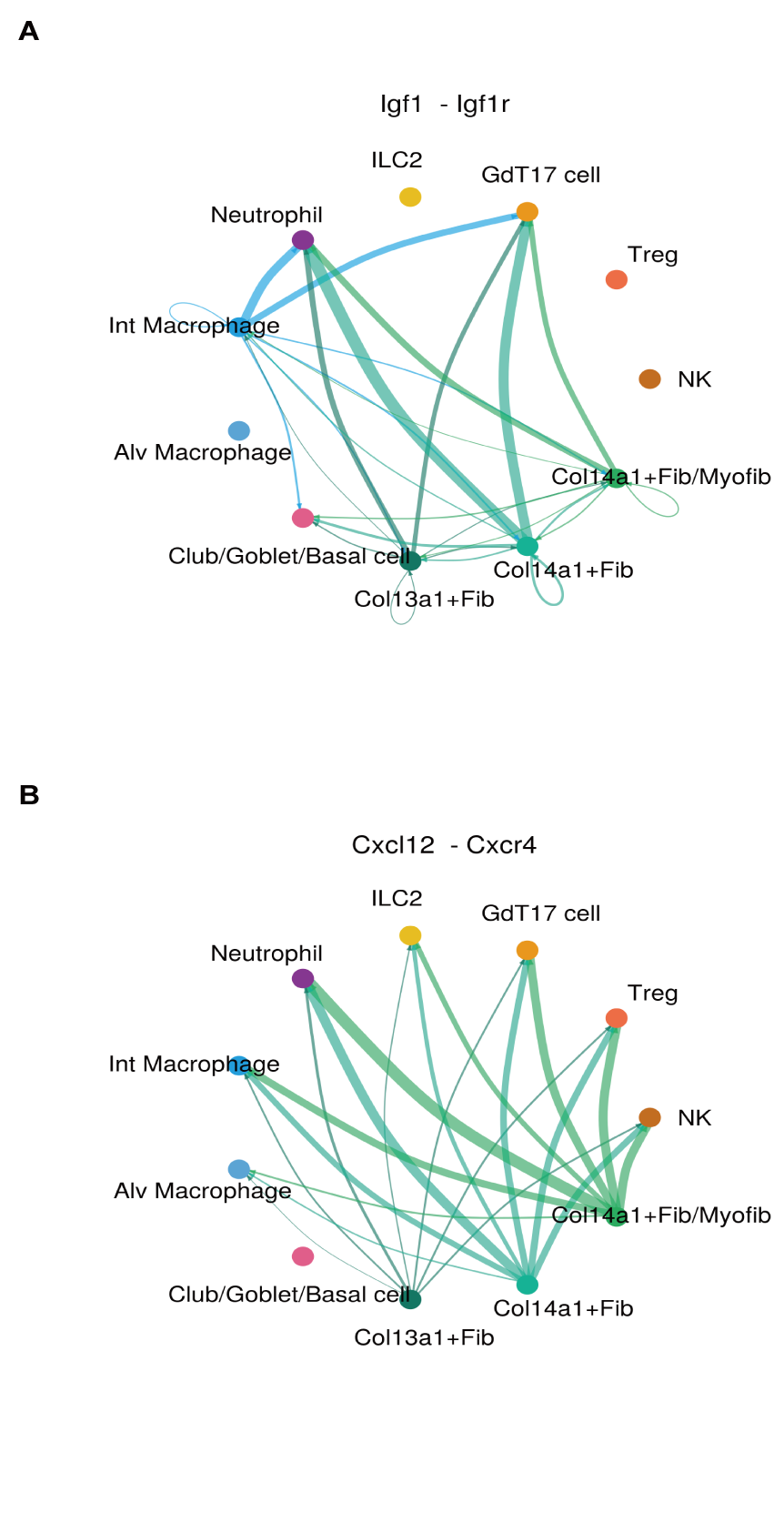

Supplement: Supplementary file 7 — Supplementary Material 7 [file 12931_2024_2706_MOESM7_ESM.tif]

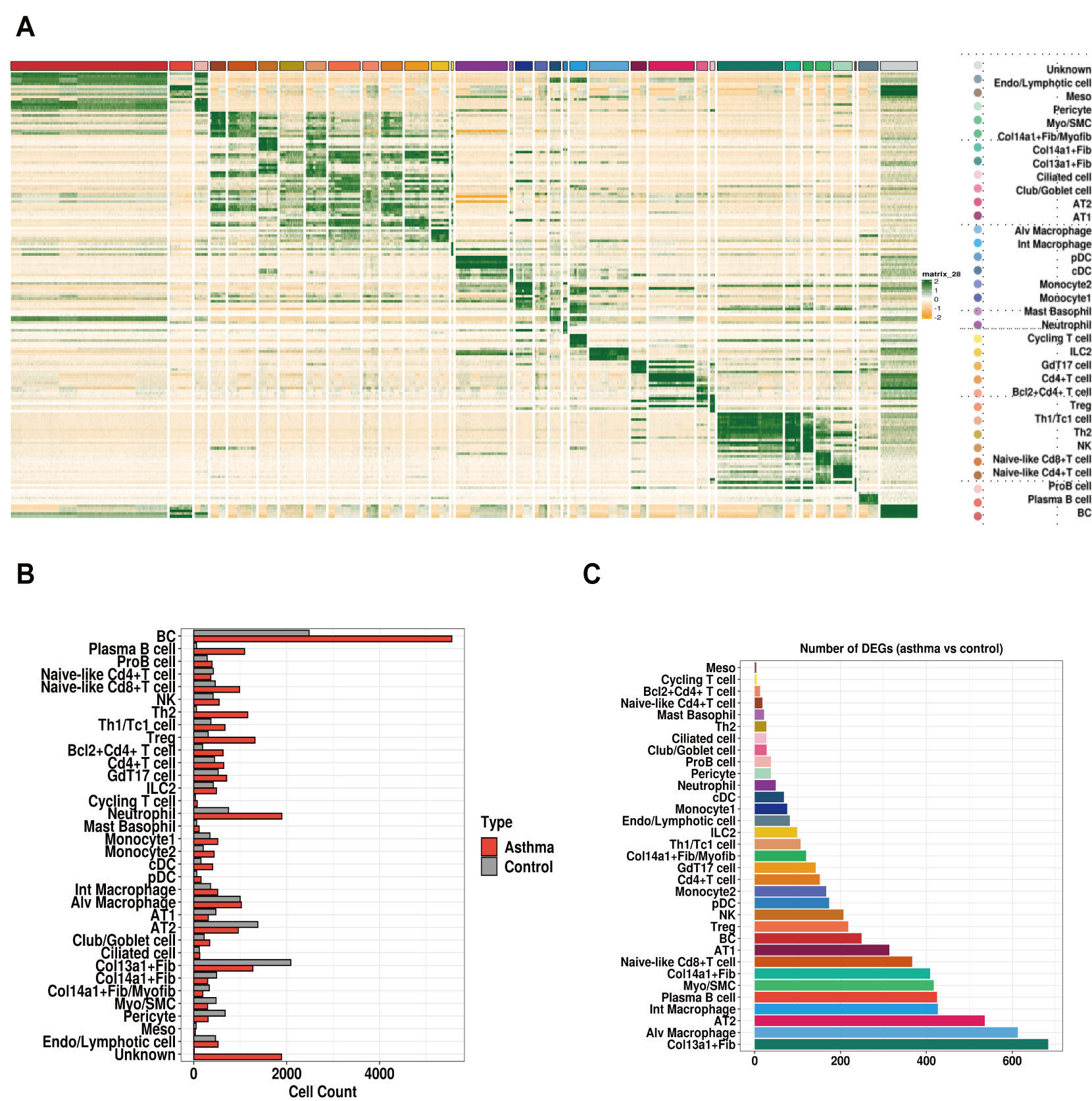

Supplement: Supplementary file 8 — Supplementary Material 8 [file 12931_2024_2706_MOESM8_ESM.tif]
