# Supplementary material for: Single-cell analysis reveals alterations in cellular composition and cell-cell communication associated with airway inflammation and remodeling in asthma
Source: Respir Res. 2024 Feb 5;25:76. doi: 10.1186/s12931-024-02706-4 (PMC10845530; doi:10.1186/s12931-024-02706-4)
Supplement: Supplementary file 9 — Supplementary Material 9 [file 12931_2024_2706_MOESM9_ESM.docx]

**Supplementary Figure Legend**

**Supplementary Fig. S1. Alterations in cell proportion and DEGs in asthma versus control group.**

**A.** Heatmap showing column-scaled expression of the top DEGs in each cell type. **B.** The barplot showing the number of cells of each cell cluster in AS and NC group. **C.** The barplot showing the number of DEGs compared between AS and NC group in each cell cluster.

**Supplementary Fig. S2. Changes in cell-cell communication network in asthma.**

**A.** Comparison of the significant ligand-receptor pairs between AS and NC groups, which contribute to the signaling pathways from macrophage subpopulations to the selected cells. Dot color represented communication probability and dot size denoted p-value. **B.** Circle plot showing inferred SPP1-CD44 pair in NC (left) and AS group (right). **C.** Circle plot showing inferred POSTN-(ITGAV/ITGB5) pair in NC (left) and AS group (right). **D.** Violin plots displaying expression level of selected genes in major sender and receiver cell clusters in NC (grey) and AS group (red).

**Supplementary Fig. S3. Specific gene expression signatures of lymphoid population.**

**A.** Feature plot of signature gene markers defining each lymphoid cell cluster. **B.** Heatmap depicts the top five most differentially expressed genes corresponding to each cluster. The expression level is indicated by the color legend.

**Supplementary Fig. S4. Specific gene expression signatures and cell proportion in myeloid population.**

**A.** Feature plot of signature gene markers defining each myeloid cell cluster. **B.** Heatmap depicts the top five most differentially expressed genes corresponding to each cell cluster. The expression level is indicated by the color legend. **C.** The cell number of each cell cluster in NC and AS group.

**Supplementary Fig. S5. Specific gene expression signatures of stromal population.**

**A.** Feature plot of signature gene markers defining each stromal cell cluster. **B.** Heatmap depicts the top five most differentially expressed genes corresponding to each cluster. The expression level is indicated by the color legend. Heatmap showing the DEGs involved in selected signaling pathways in **C.** Igf1+Fib and **D.** Myo/SMC. Log2FC and -log10 (adjusted p value) of gene were shown in color legend, enrichment score of signaling pathway was sorted and displayed in the bottom annotation bar.

**Supplementary Fig. S6. Functional analysis of target genes in the fibroblasts.**

**A.** Functional enrichment analysis revealing GO terms significantly associated with the target genes in fibroblast defined by NicheNet analysis. Color referred to the adjust p value and x-axis represents gene counts. **B.** Cnetplot showing the linkages of genes and top enriched GO terms as a network. **C.** Dot plot showing the GO-associated gene expression levels expressed in Col14a1+Fib and Col14a1+Fib/Myofib for NC and AS group, respectively. Blue and Grey indicate high and low expression, respectively.

**Supplementary Fig. S7. Functional analysis of target genes in the macrophages.**

**A.** Functional enrichment analysis revealing GO terms significantly associated with the target genes in macrophage defined by NicheNet analysis. Color referred to the adjust p value and x-axis represents gene counts. **B.** Cnetplot showing the linkages of genes and top enriched GO terms as a network. **C.** Dot plot showing the GO-associated gene expression levels expressed in Int macro and Alv macro for NC and AS group, respectively. Blue and Grey indicate high and low expression, respectively.

**Supplementary Fig. S8. Specific ligand-receptor pairs in AS.**

Circle plot showing inferred A. IGF1-IGF1R and B. CXCL12-CXCR4 pair in AS.
